# Personalized modulation of coagulation factors using a thrombin dynamics model to treat trauma-induced coagulopathy

**DOI:** 10.1038/s41540-021-00202-9

**Published:** 2021-12-07

**Authors:** Damon E. Ghetmiri, Mitchell J. Cohen, Amor A. Menezes

**Affiliations:** 1 Department of Mechanical and Aerospace Engineering, University of Florida, Gainesville, FL USA; 2 Department of Surgery, University of Colorado Anschutz Medical Campus, Aurora, CO USA; 3 J. Crayton Pruitt Family Department of Biomedical Engineering, University of Florida, Gainesville, FL USA; 4 Department of Agricultural and Biological Engineering, University of Florida, Gainesville, FL USA

**Keywords:** Dynamical systems, Systems analysis, Control theory, Biomedical engineering, Medical research

## Abstract

Current trauma-induced coagulopathy resuscitation protocols use slow laboratory measurements, rules-of-thumb, and clinician gestalt to administer large volumes of uncharacterized, non-tailored blood products. These one-size-fits-all treatment approaches have high mortality. Here, we provide significant evidence that trauma patient survival 24 h after hospital admission occurs if and only if blood protein coagulation factor concentrations equilibrate at a normal value, either from inadvertent plasma-based modulation or from innate compensation. This result motivates quantitatively guiding trauma patient coagulation factor levels while accounting for protein interactions. Toward such treatment, we develop a Goal-oriented Coagulation Management (GCM) algorithm, a personalized and automated ordered sequence of operations to compute and specify coagulation factor concentrations that rectify clotting. This novel GCM algorithm also integrates new control-oriented advancements that we make in this work: an improvement of a prior thrombin dynamics model that captures the coagulation process to control, a use of rapidly-measurable concentrations to help predict patient state, and an accounting of patient-specific effects and limitations when adding coagulation factors to remedy coagulopathy. Validation of the GCM algorithm’s guidance shows superior performance over clinical practice in attaining normal coagulation factor concentrations and normal clotting profiles simultaneously.

## Introduction

There is a dire need for targeted approaches to improve trauma patient treatment outcomes^1^. Trauma is the leading cause of death between the ages of 1–44 in the U.S.^2^; those who survive suffer huge morbidity and are left with permanent disabilities^3^. Trauma-induced coagulopathy^4^ (TIC) occurs after severe trauma and shock, is biologically characterized by perturbations to the balance between clotting and fibrinolysis^4,5^, and is clinically characterized by uncontrolled bleeding and either death or clotting complications in those who survive^6–10^. The initial traumatic hemorrhage accounts for the majority of all trauma-related deaths^11^, and 50% of the mortalities of critically-injured patients who undergo surgery^12,13^. Targeting coagulation biology and the resuscitation strategy in the first 24 h of care are critical^14^, since 80% of deaths from hemorrhage occur within this window^15^.

Current treatment involves rules-of-thumb and lab-based resuscitation guidelines. In most centers, a preset ratio of blood products is administered to rapidly control hemorrhage^11^. Although some studies attribute improved outcomes to such resuscitation control^16–19^, other studies show the opposite^20^, including conflicting data for the prehospital transfusion of fresh frozen plasma (FFP) and red blood cells^21^, and for different ratios of blood products^11,22,23^. A possible reason is the dynamic nature of patient coagulation state; too much or too little of beneficial static interventions may result in poor outcomes because of a targeting mismatch with resuscitation needs at that timepoint. While well-intentioned, blood product transfusion is linked to inflammatory morbidities and side-effects including acute respiratory distress syndrome and multi-organ failure^7,24^. Despite much research and vast improvements in clinical care, severely-injured patients that require massive transfusions still have 30% mortality^25^.

Hence, trauma patients may benefit from a tailored transfusion strategy, or from innovative treatments that include coagulation factor (blood protein) concentrates^5,6,26^. Targeting individual coagulation proteases via coagulation factor concentrates has benefit for hematologic diseases, such as hemophilia^27,28^. Although the kitchen-sink approach of using FFP in TIC has its proponents, targeted coagulation factor therapy may have better outcomes compared to FFP-based treatments^29–31^. However, some reports on modulating coagulation factors (including factor VII^32^, factor IX^33^, and factor X^34^) have shown limited benefit in individually correcting coagulopathic hemorrhage. Moreover, coagulation factor levels cannot be increased in isolation. For example, elevated levels of activated protein C (aPC) inhibit hemorrhage, but are also associated with undesirable outcomes including pneumonia, multi-organ failure, and death^35,36^. Thus, there are open requirements to: (1) confirm the benefits of modulating coagulation factors; and (2) develop a new quantitative modulation approach that incorporates interactions between coagulation factors. This article addresses these two requirements.

Existing protocols for such goal-driven trauma treatment^37–40^ use thromboelastometry, a viscoelastic coagulation assay, but this assay is time-consuming at typically about an hour per run. These protocols are non-quantitative, rely on clinical intuition and older standard procedures, and only correct for a small number of coagulation factors and/or their interactions. These protocols are qualitative because traditional statistical analysis and machine learning on static trauma patient measures like coagulation factor concentrations are not informative in diagnosing coagulation^41,42^ or treatment outcomes^43,44^. These protocols are also non-dynamic, meaning that they are unable to make time-course, patient-specific predictions of recovery, and they do not facilitate future intervention automation. A recent thromboelastogram modeling advance^45^, while promising, has yet to be deployed for dynamic treatment schemes.

**Fig. 1:**
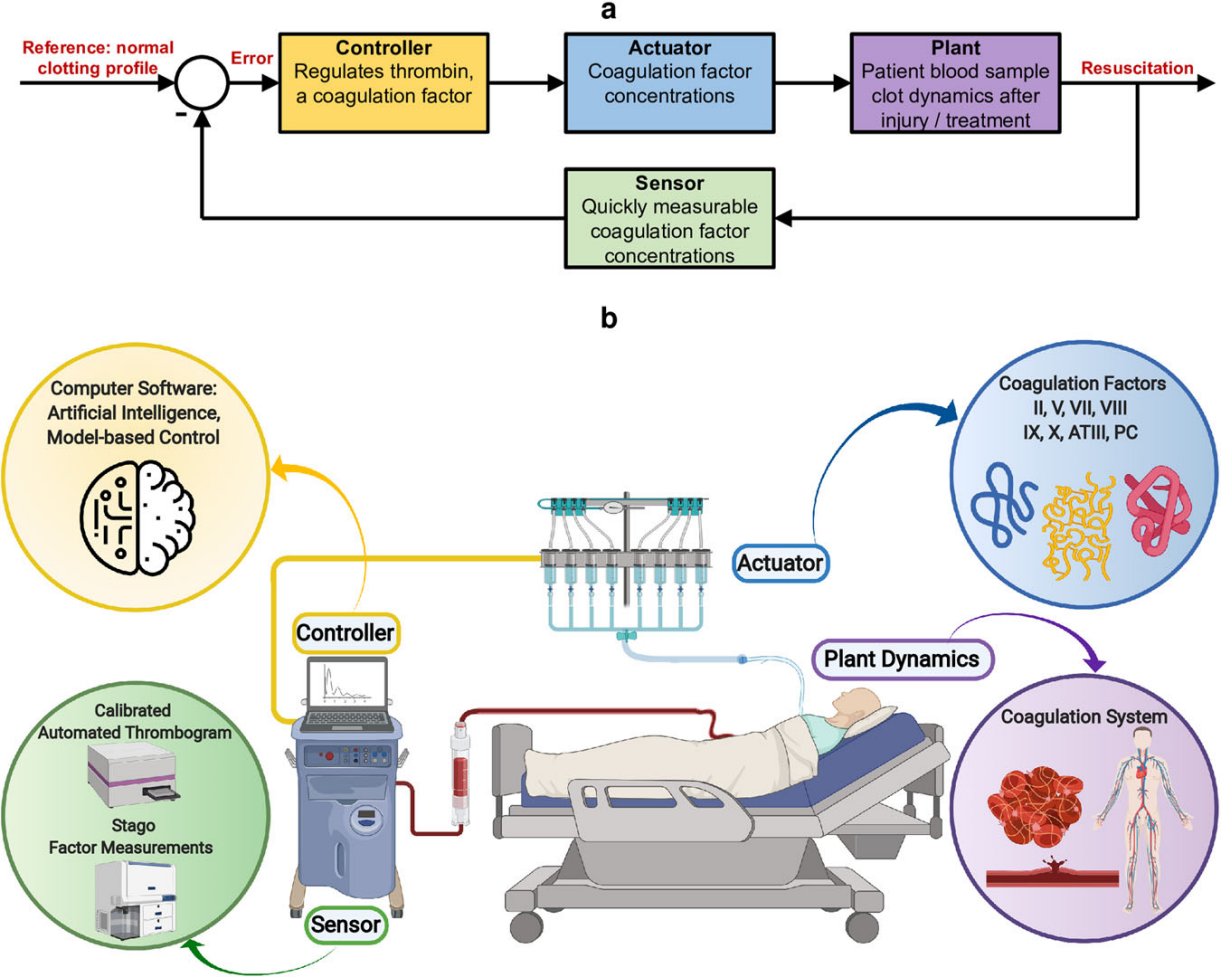
Trauma-induced coagulopathy automated treatment vision. **a** Our proposed control system architecture and block diagram. The envisioned automated system consists of four elements: sensors, a controller, actuators, and a plant, which is the patient’s coagulation process dynamics. **b** A schematic of a personalized, goal-oriented, and dynamic precision-medicine implementation to treat trauma patients at the point-of-care, which realizes the architecture in (**a**). Blood samples can be readily obtained from trauma patients. By using sensors and coagulation assays, coagulation factor concentrations in the blood sample can be quickly quantified. A smart controller algorithm then recommends a personalized treatment plan according to a goal-oriented approach, moving the patient along a recovery trajectory toward healing. This algorithm recommends coagulation factor concentrations to administer, which act as interventions to modulate patient coagulation process dynamics. This intervention approach is repeated frequently, and the treatment is adjusted dynamically. Created with BioRender.com.

Because patient responses to trauma are complex and dynamic, with risks in both hemorrhagic and thrombotic states^46^, dynamical systems approaches are preferable since they offer the ability to intervene at any timepoint, or even at multiple timepoints, in a patient’s coagulopathic trajectory. This capability can reduce a need for urgent hospital interventions to improve physiological outcomes^47^, given that there exist numerous unknown or unquantifiable priors such as patient arrival time to the hospital, injury severity, co-morbidities, and patient genetics. Dynamical systems models can capture coagulation kinetics and physiological trauma measures to improve treatment. They can also differ in how much mechanistic coagulation knowledge is harnessed^48^, or how much stoichiometry has to be included^49,50^. We seek a dynamic, goal-oriented, model-based, rapid trauma patient treatment strategy that follows the control architecture in Fig. 1a, comprising sensors, actuators, process dynamics, and a controller that uses sensed measurements of coagulation factor concentrations to actuate clotting dynamics by manipulating these concentrations. The controller vision in Fig. 1b is a point-of-care device that enables clinicians to assess, monitor, and alter trauma patient coagulation status^51^.

Toward achieving this vision, this article establishes how to quantitatively attain appropriate trauma patient treatment goals by weaving together appropriate actuators, sensors, process dynamics models, and a control algorithm into an automated treatment delivery platform that can be physically implemented at the point-of-care in future work. The original contributions of this article are as follows:

1. We provide significant evidence from patient data that there is substantial merit to administering coagulation factors to treat trauma patients. Trauma patient survival 24 h after hospital admission occurs if and only if coagulation factor concentrations equilibrate at a normal value, either from inadvertent plasma-based modulation or from innate compensation.
2. We develop a Goal-oriented Coagulation Management (GCM) algorithm, a personalized and automated ordered sequence of operations to compute and specify coagulation factor concentrations that rectify clotting. For this algorithm, we:
  - substantially improve a recent black-box process dynamics model by harnessing more data, and we then validate our improvements *in silico* on a separate dataset (Fig. 1a “plant”);
  - use rapidly-measurable coagulation factor concentrations and this updated model to predict individual clotting dynamics (Fig. 1a “sensors”);
  - confirm that administering coagulation factor concentrations accurately changes clotting as described by our improved dynamics model, noting saturating behavior for excessive coagulation factor concentration administration that motivates keeping levels between generally-accepted normal limits when modulated in a treatment scheme (Fig. 1a “actuators”); and
  - propose a novel ordering in which to tune coagulation factor concentrations to satisfy a clotting improvement goal (Fig. 1a “controller”).
3. We validate the GCM algorithm’s guidance *in silico* on a separate dataset for the critical first 24 h of care. We show superior performance over clinical practice in attaining normal coagulation factor concentrations and normal clotting profiles simultaneously.

**Fig. 2:**
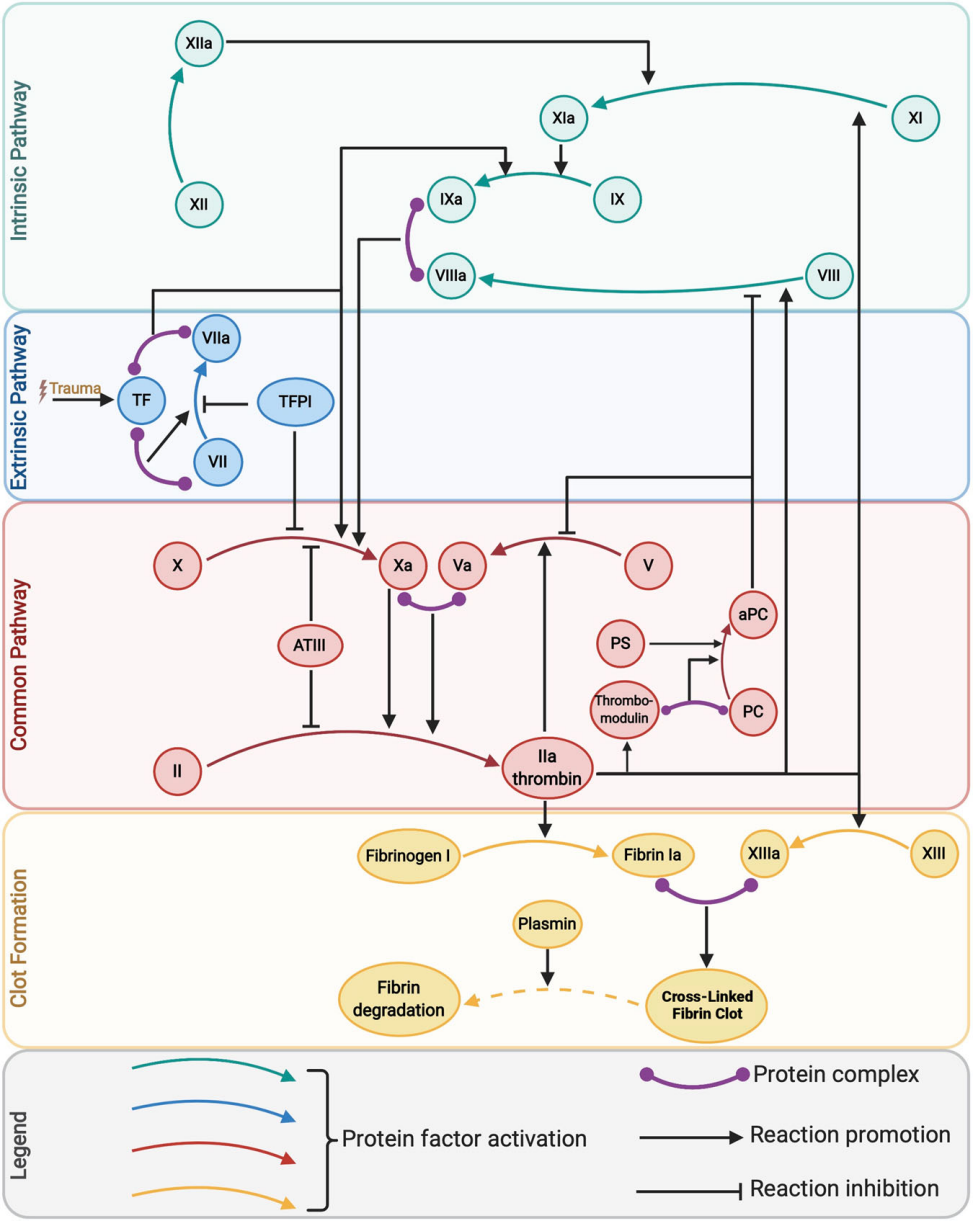
The coagulation cascade. Details of the plant, i.e., patient clotting dynamics, as embodied by the coagulation cascade, which consists of biochemical reactions that are initiated following injury. The release of tissue factor (TF) drives the process to generate thrombin, a key end product. Most of the involved proteins, called coagulation factors, are denoted by Roman numerals. An added letter “a” indicates activation. Anticoagulant proteins include tissue factor pathway inhibitor (TFPI), antithrombin (ATIII), protein C (PC), and protein S (PS). Created with BioRender.com.

Coagulation factors are central to our control approach: they are the actuators for trauma patient treatment, and their concentrations can be rapidly measured within a few minutes using sensors and coagulation assays. The process of clot formation after injury proceeds according to the biochemical kinetics of the coagulation cascade^52^, Fig. 2, driven by coagulation factor concentrations. The resultant dynamics constitute the plant in Fig. 1a, b.

Thrombin, factor IIa, is the end product of the coagulation cascade, and thrombin generation measures can be leveraged to predict hemostatic potential and transfusion requirements^53^. Such measures can replace conventional coagulation tests like prothrombin time (PT), partial thromboplastin time (PTT), international normalized ratio (INR), and platelet counts, all of which have limitations^54^. Thrombin is a unique protein that functions as both a procoagulant and an anticoagulant^55^. As a procoagulant, thrombin activates platelets, converts fibrinogen into strands of fibrin, effects the cross-linking of fibrin to produce a firm fibrin clot by activating factor XIII, and catalyzes other coagulation-related reactions, like the activation of factors V, VIII, XI, and protein C (PC), which in turn regulate thrombin generation^56^. As an anticoagulant, thrombin binds to thrombomodulin, a receptor protein on the endothelial membrane of a blood vessel, initiating a series of reactions that leads to fibrinolysis^55^. Thrombin’s activation of PC, a strong anticoagulant implicated in TIC, has also been extensively studied^35,57^ and is therefore included in our study.

The calibrated automated thrombogram (CAT)^58^ is a coagulation assay that can measure the concentration time-history of thrombin in a plasma sample. However, this assay takes about 45–60 min to run, without including plasma sample preparation time. Such delays are far too long to be used at the bedside to predict and guide treatment and outcomes^59^. Models^48^ that mathematically predict the concentration time-history of thrombin from patient plasma sample coagulation factor concentrations, and that thereby capture the dynamics of the coagulation system process while simultaneously replacing the CAT assay, can be useful in controller development. Among the few existing thrombin-prediction models, either important coagulation factors like PC are excluded^48^, computational loads are high^60^, or simulation results are not thoroughly validated against experimental data^61,62^. These deficiencies are addressed in this article. As we also show, a treatment algorithm that leverages such an improved model can provide frequent, personalized, and dynamic recommendations based on sample clotting predictions, to move a trauma patient’s coagulation state toward a desired recovery trajectory.

## Results

### The merit of administering coagulation factors to personalize trauma treatments

Trauma patient therapy does not yet use quantitative coagulation factor concentration guidance, possibly because common static machine learning approaches on typical patient data with coagulation factor concentrations are uninformative^41–44^. We highlight this fact for the 1671-patient Activation of Coagulation and Inflammation in Trauma (ACIT) dataset, a previously described^63^ prospective cohort study of severely-injured trauma patients who were admitted to a Level I trauma center (Supplementary Fig. 1 overviews the numbered datasets used to develop and validate all results in this article). In this dataset, dataset 1, and throughout this article, coagulation factor concentrations are measured using the STA Compact Max^®^ device (Stago), which reports these concentrations in units of percent activity, a measure that is with respect to the normal coagulation factor concentration in a healthy person. Supplementary Fig. 2 shows the lack of correlation between coagulation factor concentration measurements and patient measures such as age, injury severity score (ISS), PTT, and INR. Unsurprisingly, Supplementary Fig. 3 shows that coagulation factor concentration measurements are uncorrelated with injury severity, and are hence not useful for classification to elicit ISS. Additional static machine learning results, such as those from a bilayered neural network, Supplementary Fig. 4a, and from a support vector machine, Supplementary Fig. 4b, fail to predict patient mortality much better than a coin flip. Even dynamic machine learning is not satisfactory^44^.

Moreover, initial biomarker and injury measurements are not correlated to treatment received, and so cannot predict resuscitation need and adverse outcomes. This unpredictability is illustrated by 252 of the 1671 trauma patients who survived the first 24 h and for whom we had complete data, dataset 2 (Supplementary Fig. 5 has demographic information for this patient subset), as well as the 96 patients who died within the first 24 h, dataset 3. We found that the means of trauma patient coagulation factor concentrations do not indicate if a trauma patient is at high risk for mortality within 28 days (Supplementary Fig. 6a), or at high risk for massive transfusion (Supplementary Fig. 6a) or a thrombotic event (Supplementary Fig. 6b). Equally important, coagulation factor concentrations are uncorrelated to treatment and resuscitation: trauma patients who receive FFP, no matter the number of units they receive, show substantial variation in coagulation factor concentration changes over time (Supplementary Fig. 7), potentially due to a lack of characterization of, and inherent variability in, coagulation factor concentrations per FFP unit. Therefore, FFP units are not predictive of increases or decreases in coagulation factor concentrations. This also substantiates why FFP administration has mixed results for treatment, since units may not deliver required coagulation factors or may oversupply unnecessary coagulation factors in different patients at different timepoints.

**Fig. 3:**
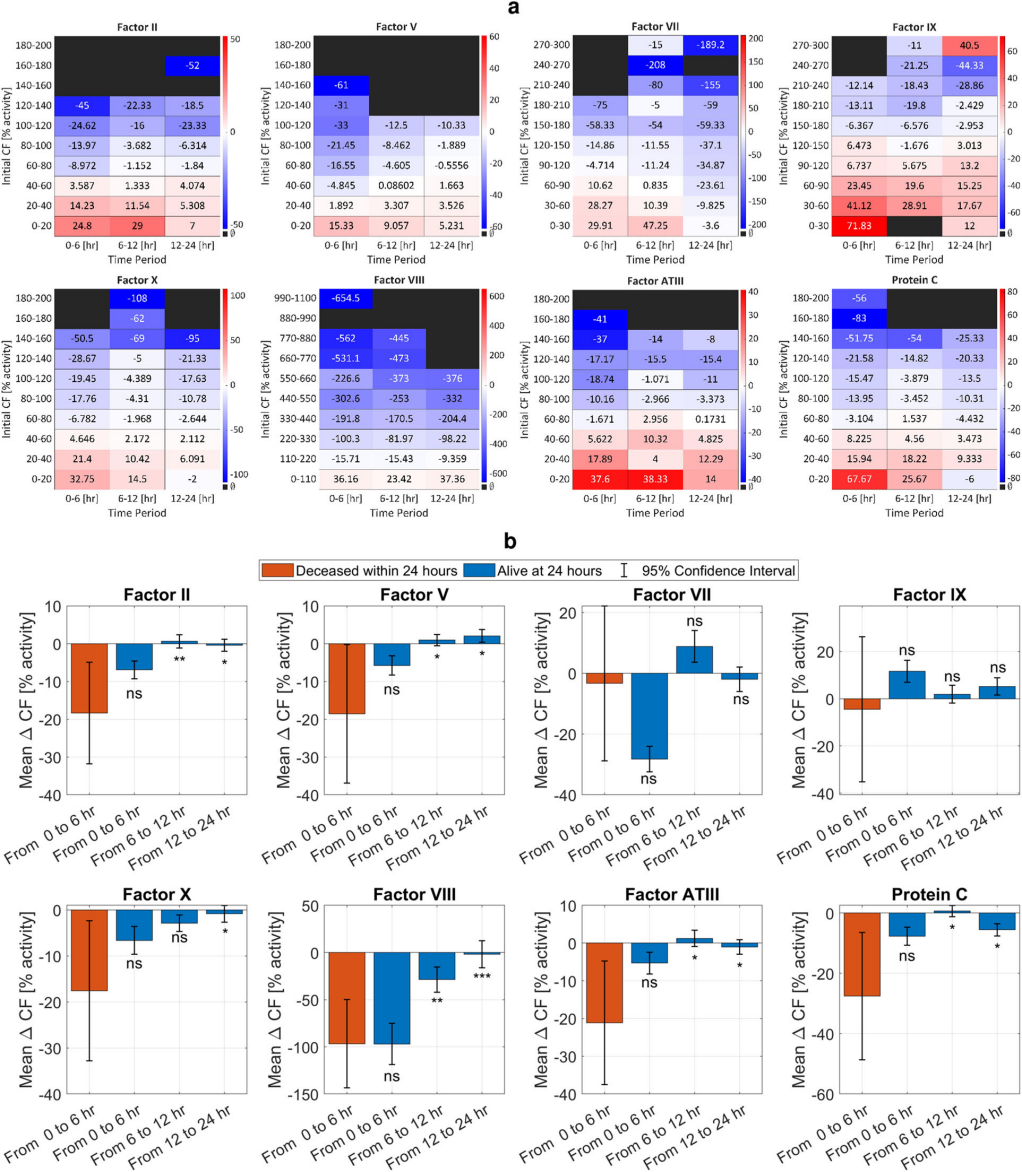
Dynamical and statistical analyses of changes in coagulation factor concentrations over time. **a** Heatmap of changes in concentrations of coagulation factors (CFs) for 252 trauma patients who survived 24 h, dataset 2, grouped by initial concentration (percent activity) at the beginning of time period, show that concentrations move toward an equilibrium over time. If the starting concentration of any of factors II, V, VII, VIII, IX, X, ATIII, and protein C is low then the concentration increases, and if the starting concentration is high then the concentration decreases. Numerical values in the cells indicate the mean change of CF in that group, and the cell color represents this mean ΔCF according to the color bar on the right. **b** Comparison between the mean of coagulation factor concentration changes in trauma patients who died after 6 h, dataset 3, to the mean of coagulation factor concentration changes in the 252 trauma patients of panel (**a**) who were alive after the first 24 h, dataset 2, at different time periods (from 0 to 6 h, from 6 to 12 h, and from 12 to 24 h). The bar value indicates the mean of each group, the error bar represents a 95% confidence interval, and the *p*-value significance is indicated above/below each bar (ns: not significant, *p* > 0.05; *p ≤ 0.05; **p ≤ 0.01; and ***p ≤ 0.001). This panel confirms that patients who recover have coagulation factor concentrations that move to an equilibrium, with the change in coagulation factor concentrations moving to zero.

Nevertheless, a close examination of the changes in coagulation factor concentrations for subgroups of the 252 survivor trauma patients based on initial coagulation factor levels shows clear dynamic information over the first 24 h after hospital admission. We illustrate these dynamics using a heatmap of changes (Δ) in coagulation factor (CF) concentrations at different time periods in the first 24 h (0 h–6 h, 6 h–12 h, 12 h–24 h), Fig. 3a. These changes are computed by subtracting the coagulation factor concentration at the period end time from the coagulation factor concentration at the period start time. For each period, the changes are arranged into heatmap cells according to the coagulation factor concentration at the start of the time period. The mean ΔCF is the number displayed in each heatmap cell, and is matched to an appropriate color.

Specifically, coagulation factor concentrations move toward an equilibrium concentration that is representative of homeostasis: concentrations that start from a low value increase over time, while concentrations that start from a high value decrease over time, Fig. 3a. This observation holds true for all coagulation factors. In general, we see darker colors at the lower and upper ends of the Fig. 3a heatmaps at the start time (left side), indicating a sharper change in coagulation factor concentration over the first time period. As coagulation factor concentrations move toward equilibrium over time, the magnitude of these changes decrease, and we observe white and lighter color shades (right side of the heat maps).

To test the significance of our observation that coagulation factor concentrations move toward an equilibrium in patients who recover, we performed a hypothesis (*p*-value) test that contrasted coagulation factor concentration changes in patients who survived to those who died in the first 24 h. We defined four groups: (1) for patients who died between 6 and 24 h, their changes in coagulation factor concentrations between 0 and 6 h (Deceased 0–6 [h]); and for patients who were alive at the 24 h mark post hospital admission time (Alive), their changes in coagulation factor concentrations between (2) 0 and 6 h, (3) 6 and 12 h, and (4) 12 and 24 h. We performed Welch’s *t*-test and calculated *p*-values for *α* = 0.05. The null hypothesis (*H*_0_) was that the mean change in a coagulation factor’s concentration is equal for patients who are dead or alive, i.e., *μ*_*x*_ = *μ*_*y*_, where *μ*_*x*_ and *μ*_*y*_ are the deceased and alive sample means, respectively.

Fig. 3b is a barchart representation of the mean of coagulation factor concentration changes over different time windows, with error bars that indicate a 95% confidence interval. For all coagulation factors, there is no significant difference in concentration changes between the two groups (deceased and alive) from 0 to 6 h, because the two groups had similar initial conditions. However, in the later time periods in patients who survived, i.e., from 6 to 12 h and from 12 to 24 h, there is a significant difference in the mean coagulation factor concentration change of survivors compared to the deceased. The exceptions are for factors VII and IX, due to the large variability of these coagulation factors in the deceased. The results of this analysis reject the null hypothesis and therefore favor an alternative hypothesis *H*_*a*_ of non-equal means, i.e., our results indicate that there is enough statistical evidence to conclude that mean changes in coagulation factor concentrations of patients who recovered are significantly different from those of patients who died.

**Fig. 4:**
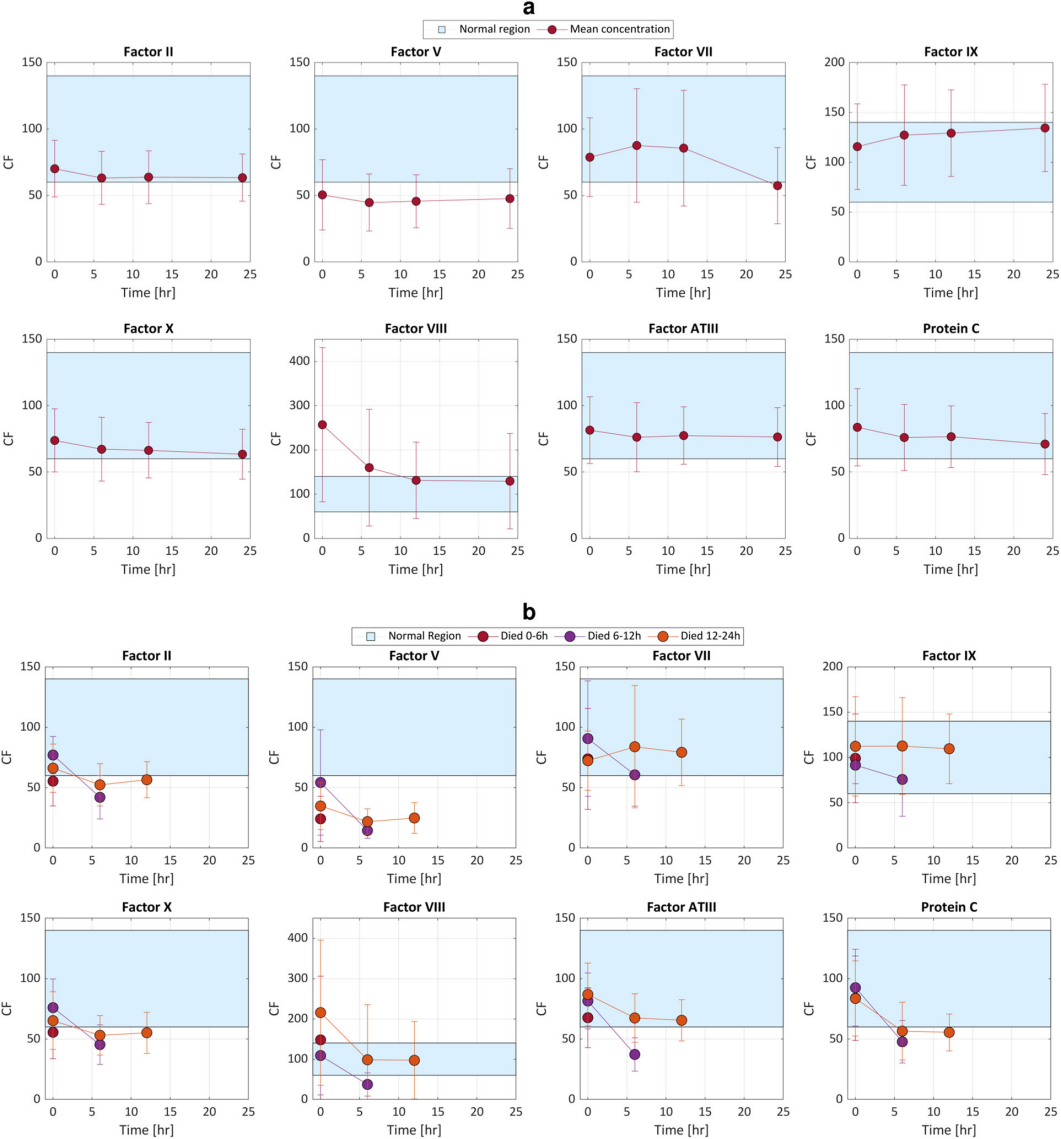
Trauma patient coagulation factor concentration time history over the first 24 h. **a** Mean ± one standard deviation of the concentrations of coagulation factors (CFs) during the first 24 h after hospital admission, for factors II, V, VII, VIII, IX, X, ATIII, and protein C of 252 trauma patients, dataset 2 (demographics in Supplementary Fig. 5a). On average, the coagulation factor means converge to normal, where normal is the coagulation factor concentration range of 60–140% activity. **b** Mean ± one standard deviation of the CF concentrations for another 96 patients that died in the first 24 h after hospital admission, dataset 3, grouped by mortality time window. On average, the coagulation factor means converge outside normal. Units of coagulation factor concentrations are reported as percent activity.

Given that patients who survive the first 24 h have coagulation factor concentrations that converge to equilibrium values (either from inadvertent plasma-based modulation of coagulation factor concentrations, or from innate coagulation factor compensation), Fig. 4a shows that these equilibria are within normal ranges of 60–140% activity^64,65^. Moreover, Fig. 4b shows that trauma patients who die between 6 and 24 h have coagulation factor concentrations that also converge to equilibrium values, but these are outside normal ranges. It follows that our data support the claim that a necessary and sufficient condition for trauma patients to survive the first 24 h is to administer coagulation factors such that their concentrations will equilibrate at a normal value. The necessary condition is Fig. 4a, and the contrapositive of the sufficient condition is Fig. 4b. Consequently, there is merit to correcting individual coagulation factors dynamically over time, tailored to each patient to improve treatment outcome.

### Improved prediction of process dynamics from coagulation factors

Since there is merit to administering coagulation factors, the next question is how to administer them to personalize trauma patient treatment. Predictions of effect are first required. Menezes et al. proposed a third-order linear dynamical systems model^48^ to rapidly predict CAT trajectories from quickly-measured coagulation factor concentrations. While this model has satisfactory prediction capability, we hypothesized that an embedded constraint limits its prediction accuracy. We investigated whether model improvement was possible without changing model structure, by just adding a single degree-of-freedom parameter to remove this underlying constraint.

We examined the input–output model

1 $$\frac{Y(s)}{U(s)}=\frac{{K}_{n}}{{s}^{3}+{K}_{2}{s}^{2}+{K}_{1}s+{K}_{0}}{e}^{-{K}_{d}s},$$

where *K*_0_, *K*_1_, *K*_2_, *K*_*n*_, and *K*_*d*_ are five patient-specific model parameters (the prior model^48^ used four parameters with its fifth parameter constrained; the models are mathematically-equivalent), *Y*(*s*) is the predicted output thrombin concentration time-history in the frequency domain, and *U*(*s*) is a 5 pM impulse input tissue factor (TF) concentration in the frequency domain. An impulse input is an input signal with a very high magnitude that is applied to a system over a very short time^66^. Theoretically, this magnitude approaches infinity as time goes to zero. In practice, this magnitude is taken to be some finite value, commonly 5 pM in the CAT literature, a value that also has experimental justification^48^. Practically, the CAT is instantiated with 5 pM of TF in the plasma sample, which then rapidly depletes.

**Fig. 5:**
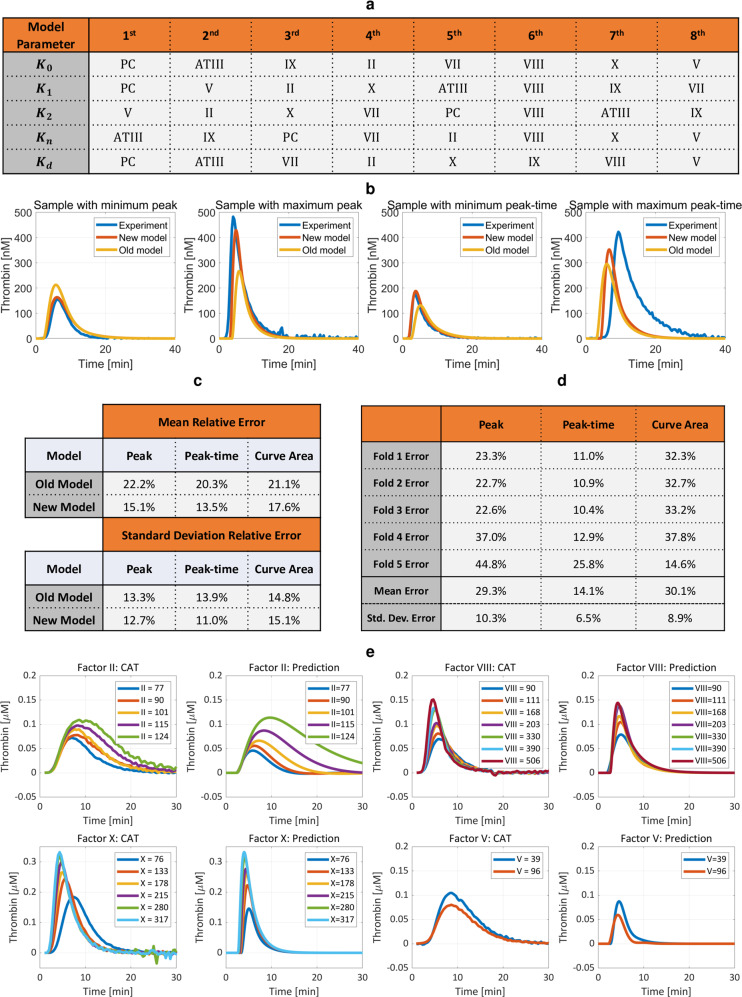
Our new validated thrombin concentration time-history model has better predictive performance compared to the previous model. **a** Sorted correlation of coagulation factor concentrations to model parameters, from highest to lowest. Activated PC is the main driver for three of five parameters in the improved model. **b** Thrombin concentration time-history prediction is much improved compared to an older model^48^, shown here for four edge cases of minimum peak, maximum peak, minimum peak-time, and maximum peak-time. **c** Mean percent error of three CAT parameters estimated using the old model and the new model for 40 trauma patient samples, dataset 5. Percent errors are calculated for each sample by comparing each CAT parameter estimated using dynamic models to the actual CAT parameters from fits of experimental data, and then the mean and standard deviation of all sample relative errors reported. For example, $\,{{\mbox{CAT Peak percent error}}}=| \frac{{{{\mbox{Peak}}}}_{{{\rm{model}}}}-{{{\mbox{Peak}}}}_{{{\rm{experiment}}}}}{{{{\mbox{Peak}}}}_{{{\rm{experiment}}}}}| \times 100$. **d** Five-fold cross-validation bootstraps the data (datasets 4 and 5) and confirms that model predictions are valid with acceptable mean percent error. **e** CATs predicted with our improved model using an additional experimental dataset^48^, dataset 8, that was not harnessed for learning. Our model is able to accurately capture both trends and magnitudes of actual CATs. Numbers in the legend indicate coagulation factor concentration reported as percent activity.

We included the initial PC concentration with the initial concentrations of factors II, V, VII, VIII, IX, X, and antithrombin (ATIII), creating new linear regressions for the five parameters via the same greedy method, the matching pursuit algorithm^67^, as previously^48^. The important role of PC in the coagulation cascade^68^ as described by our extensive work ^35,57,69^ motivated this modification. We found that our model updates substantially improved CAT predictions. On a dataset of 60 samples (20 individual healthy donors and 40 trauma patients, datasets 4 and 5 in Supplementary Fig. 1)^48^, we applied stepwise linear regression that consists of sequentially and greedily adding the linear effect of a coagulation factor concentration measurement that most reduces the error of a least-squares fit to all data for each of thrombin model (1) parameters. The coagulation factor that minimizes this least square error has the greatest contribution to the system dynamics captured by that particular model parameter. The stepwise process was repeated until further linear additions of coagulation factor concentration measurements no longer improved the fit. The order of these coagulation factors for each model parameter is presented in Fig. 5a. This figure confirms the importance of PC and its prime effect on three of the five model parameters.

Visual comparisons of model improvement are in Fig. 5b, for four edge cases of minimum peak, maximum peak, minimum peak-time, and maximum peak-time. For trauma patients, the mean peak error improved to 15.1% from 22.2%, the mean peak-time error improved to 13.5% from 20.3%, and the mean thrombin potential (area under the CAT curve) improved to 17.6% from 21.1%, Fig. 5c. Fig. 5a shows that the model fitness improvement of Fig. 5b, c is not because of information increase from adding another coagulation factor to an existing list, but rather because protein C is the most impactful dynamics contributor.

We validated our model in two ways, first with five-fold cross-validation, and second on a separate dataset that was not used for training. Five-fold cross-validation^70^ bootstraps available data by subdividing it so that 80% is used for training and the remaining 20% is used for validation. The process is iterated five times for five unique divisions (folds) of the original dataset. The mean model output properties of these five iterations for the combined dataset of 20 normal samples and 40 trauma patient samples (datasets 4 and 5) are reported in Fig. 5d. This figure confirms good prediction capability. Obtaining errors of 20% or less is a rule-of-thumb for mechanical systems, with less than 10% the ultimate goal through model refinement^71^; given significant inherent biological variability compared to mechanical systems and possible as-yet-undiscovered interactions, a target of 30% or less error is not unreasonable. We anticipate that model prediction will improve with more trauma CAT data.

Additional model validation was accomplished with a separate validation dataset, dataset 8, that was not used for model training. This validation set started with normal plasma samples that had coagulation factor concentration and CAT measurements, and into which were spiked increasing concentrations of factors II, VIII, and X that were then quantified. Our model trained on the separate 60 samples (datasets 4 and 5) can predict the 20 experimental validation CATs (dataset 8) almost perfectly, Fig. 5e.

### Effects and limitations of coagulation factors as actuators

To examine the dynamic modulation effects of coagulation factors, we used experimental datasets 4, 5, and 7 in Supplementary Fig. 1. Dataset 7 started with normal plasma samples that had coagulation factor concentration and CAT measurements, and into which were spiked increasing concentrations of factors II, VIII, and X. New coagulation factor concentration and CAT measurements were taken after each spike.

We examined the effects of different initial TF concentrations and coagulation factor concentration spikes on system poles. The poles of a dynamical system are characteristic parameters that determine the system’s stability and output response^66^. These poles can be obtained from a transfer function model of a system by determining the values for which the denominator of the transfer function becomes zero, i.e., we find the poles of a trauma patient’s coagulation system by setting the denominator of model (1) to zero and solving the resultant equation for *s*.

**Fig. 6:**
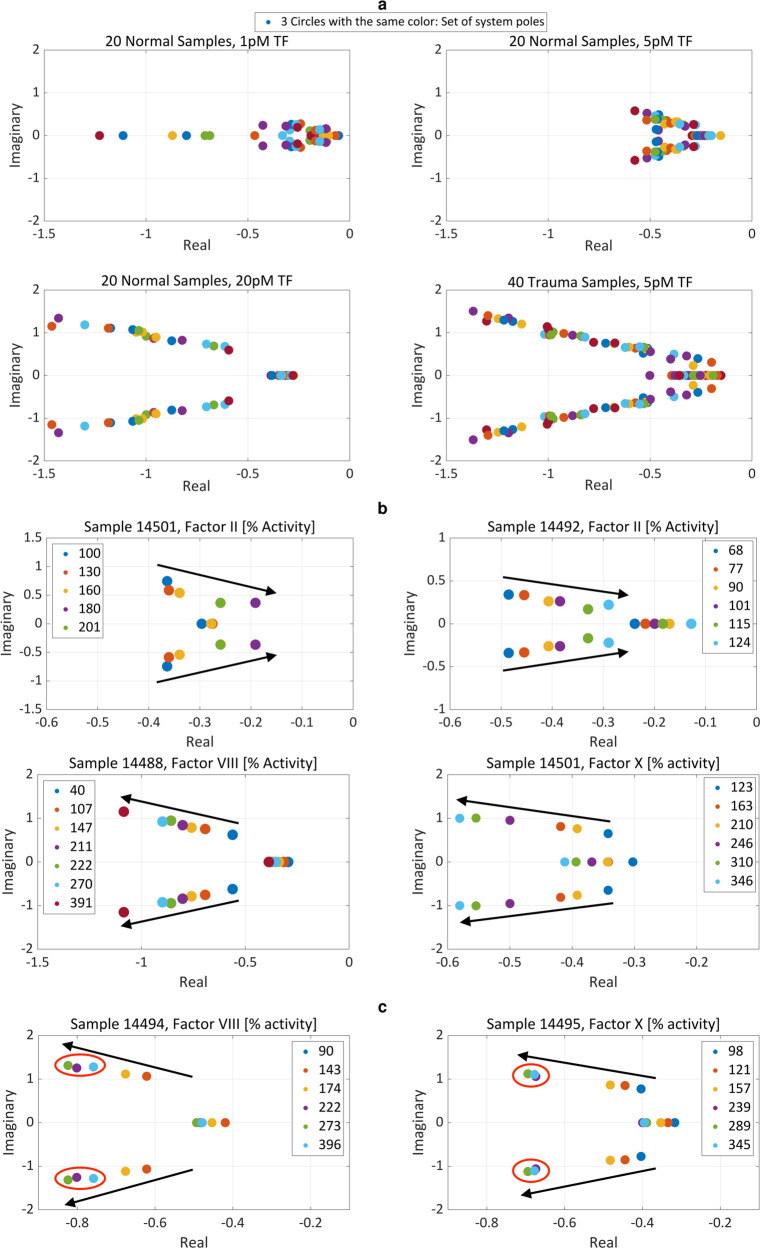
Each coagulation factor has a unique effect on system dynamical behavior as described by the movement of pole locations, and is often accompanied by nonlinear limitations, for instance, saturation. The dots in each panel show complex plane pole locations for the transfer function (1) fitted to experimental CATs using the MATLAB Simulink Design Optimization (SDO) toolbox. The three poles of each fit are shown with the same color. **a** Pole locations of the fitted transfer functions for 20 normal plasma samples, dataset 4, with inputs of 1 pM TF, 5 pM TF, and 20 pM TF; and 40 trauma patient plasma samples, dataset 5, each with an input 5 pM TF. Higher initial TF concentrations move poles away from the origin, and higher initial TF concentrations in normal samples replicate the effects of trauma. **b** Increasing the concentration of factor II in two normal plasma samples moves system poles toward the origin, while increasing the concentration of factors VIII and X in normal plasma samples moves poles away from the origin. **c** Saturation in pole movement is evident for increasing concentrations of factors VIII and X in normal plasma samples. For **b**, **c** numbers in the legend indicate coagulation factor concentration reported as percent activity.

Surprisingly, for 20 normal plasma samples from different donors, we found that increased initial TF concentration caused substantial system pole movement away from the origin, essentially recapturing trauma patient variability, Fig. 6a. That is, trauma effects are replicable by manipulating TF concentration. Similarly, as Fig. 6b and c show, increases in the concentration of factor II in normal plasma samples pushed coagulation system poles toward the origin, while higher levels of factors VIII and X caused system poles to move away from the origin. Physical limitations like saturation are also apparent in some normal plasma samples, Fig. 6c, with additional increases in coagulation factor concentrations beyond a certain value not impacting system behavior. We hypothesize that this observed result is due to the limiting availability of other coagulation factor concentrations that form complexes in the system.

The results of spiking isolated coagulation factors into validation plasma samples, dataset 8 and Fig. 5e, also validate the actuator effect of each coagulation factor on the human coagulation system and thrombin generation. The isolated increase of each coagulation factor concentration results in a unique change in thrombin profile properties. For example, an increase in factor II leads to an increased peak and increased curve area, an increase in factor VIII mostly only affects the peak value, and an increase in factor X increases peak value and simultaneously reduces peak-time. These effects can be harnessed by an algorithm that seeks to make a thrombin profile more normal, next.

### Personalized control of trauma patient thrombin dynamics using coagulation factors

We determined a target goal CAT and an associated region inside which any CAT trajectories can be considered normal by calculating the maximum, minimum, and mean of the experimental data at each time point for all normal plasma samples, dataset 4 (Supplementary Fig. 8) and fitting model (1) to this data. To evaluate how well the identified region represented normal, we validated it using five normal samples, dataset 9, that were different from dataset 4, which was used for identification. We contrasted the CAT profile of these five validation samples against the normal region (Supplementary Fig. 9) using mean relative error (MRE), the mean of the error at each time point where the profile was not within normal minimum and maximum bounds. MREs for dataset 9 are reported in Supplementary Table 1.

**Fig. 7:**
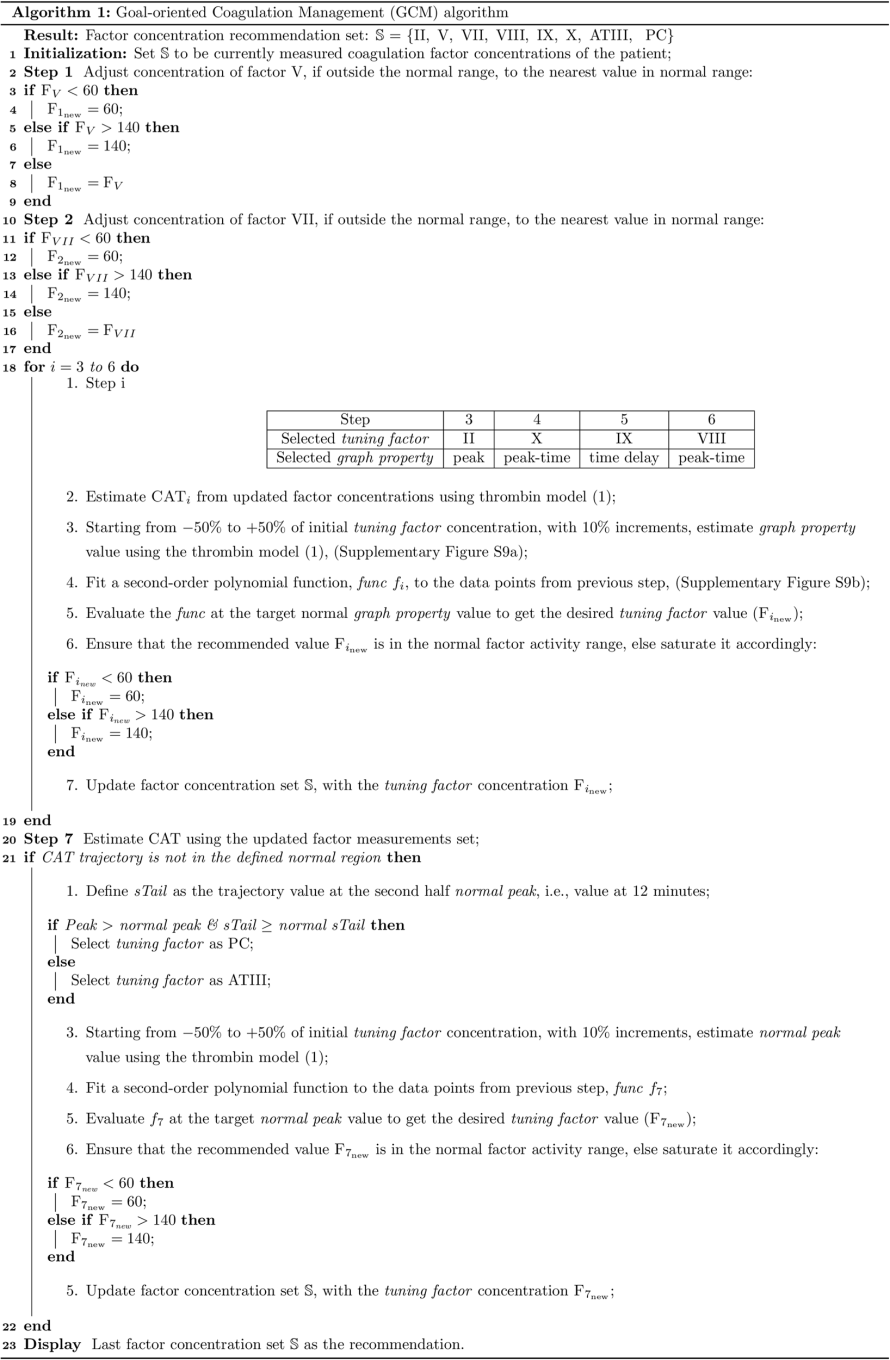
The proposed GCM algorithm enables frequent, dynamic, and personalized TIC treatment. This algorithm systematically recommends coagulation factor concentrations to move a patient CAT trajectory toward normal, while also maintaining concentration values within normal activity ranges. This algorithm serves as the controller block in Fig. 1a.

**Fig. 8:**
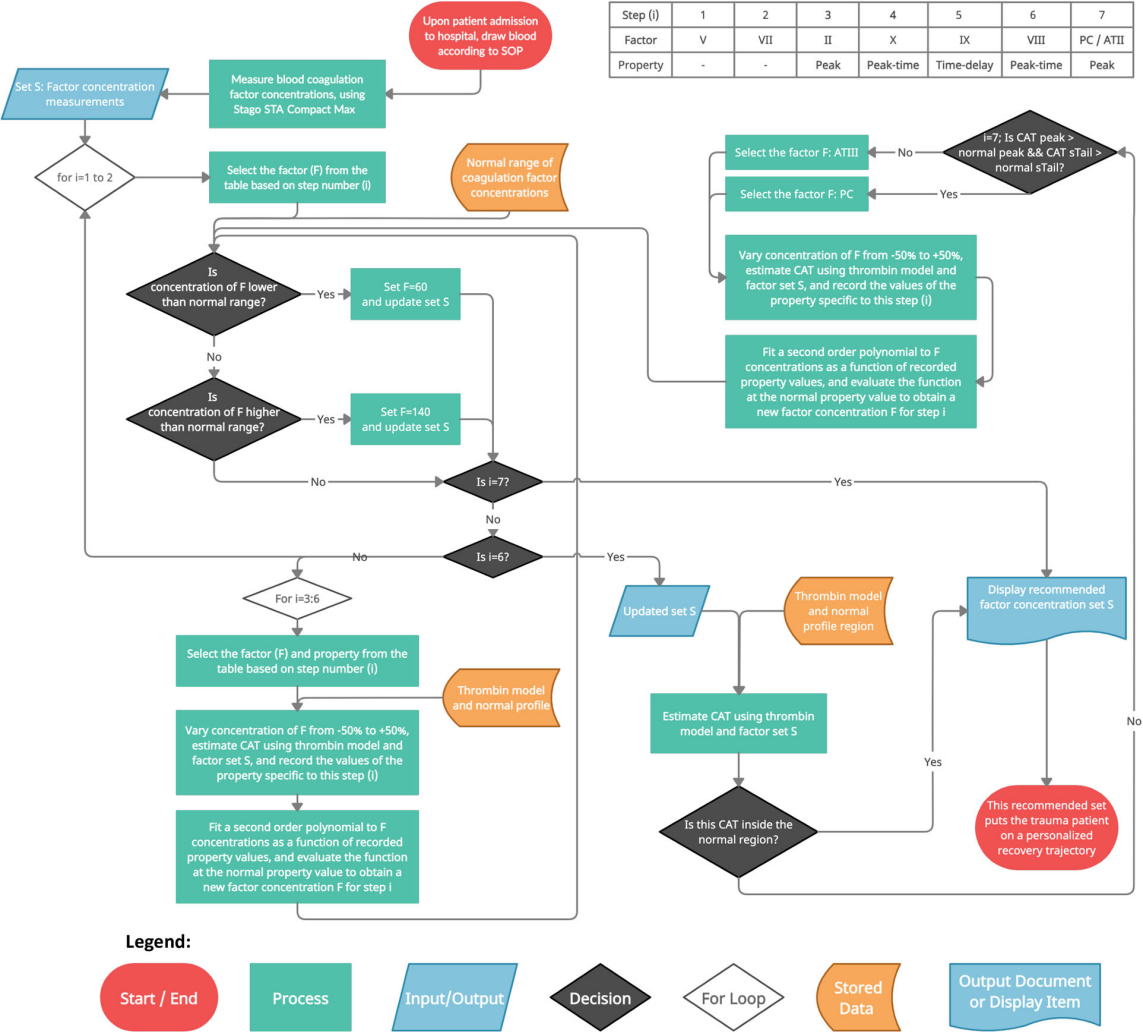
Flowchart of the proposed GCM algorithm that enables frequent, dynamic, and personalized TIC treatment. This algorithm systematically recommends coagulation factor concentrations to move a patient CAT trajectory toward normal, while also maintaining concentration values within normal activity ranges. This algorithm serves as the controller block in Fig. 1a.

We then developed a Goal-oriented Coagulation Management (GCM) algorithm, Figs. 7 and 8, to recommend a personalized set of coagulation factor concentration changes to move trauma patients onto a recovery path. Our algorithm harnesses CAT estimates from coagulation factor concentration measurements via model (1), and identifies a patient-specific mapping, Supplementary Fig. 10, from coagulation factor concentration changes to thrombin clotting effects according to these CAT estimates rapidly and in real-time. This mapping is a simple second-order polynomial, justified by the Akaike Information Criterion^72^ as being the smallest-parameter fit that is the most-informative.

We defined algorithm treatment goals to simultaneously (a) move coagulation factor concentration values toward normal equilibrium values, and (b) achieve a normal thrombin (clotting) profile. To attain these treatment goals, the sequence of GCM algorithm operations was developed by:

(i) Prioritizing reaching a normal range of coagulation factor concentrations;
(ii) Ordering how four thrombin profile properties mimic normal clotting; and
(iii) Investigating the isolated effects of coagulation factors on thrombin profile properties.

To satisfy (i), our algorithm first corrects concentrations of coagulation factors that have minimal impact on thrombin profile. Thereafter, the estimated CAT is progressively corrected by modulating a coagulation factor concentration according to our new modeled dynamic interactions, with the updated concentration checked to be in the normal range at the end of each thrombin profile correction step. For (ii), we set the order in which the algorithm tunes CAT properties as follows: thrombin generation (peak), response time (peak-time), time delay in system response (time delay), and thrombin potential (area under the curve, which is evaluated and compared using the profile tail, called “sTail”). For (iii), we had investigated the most impactful individual coagulation factor concentration changes on estimated CAT properties by performing numerical simulations on datasets 4 and 5 (Supplementary Fig. 10). We determined the coagulation factors that have primary and secondary impact on each thrombin profile property, and our algorithm tunes these coagulation factors to adjust a predicted CAT property in each algorithm step.

Our GCM algorithm first modulates the concentrations of factors V and VII into their normal range because these coagulation factors have limited impact on CAT estimates according to our improved thrombin dynamics model, and because their small effects can be overcome by changes in the remaining coagulation factor concentrations as the algorithm progresses. Next, overcoming a trauma patient’s thrombotic or hemorrhagic condition is imperative, equivalent to manipulating a CAT’s peak value. Hence, the algorithm next changes the concentration of thrombin precursor factor II, thereby changing the predicted CAT peak as much as possible while maintaining this coagulation factor’s concentration inside its normal range. Factor X is corrected thereafter, to supplement the peak correction effect of factor II that may be saturated at a normal limit, and also to compensate for changes in peak-time that are caused by factor II manipulation because factor X’s peak-time effect is opposite that of factor II. Factor X also affects the CAT time-delay, which can then be rectified by adjusting the concentration of factor IX with little effect on CAT peak. Modulating factor VIII follows, because changing this coagulation factor allows for fine control of peak-time with minimal effect on CAT peak or time-delay.

The final step of the GCM algorithm ensures that the recommended CAT estimate is inside the normal region. If not, then the algorithm chooses to manipulate one of two anticoagulant factors, either protein C or ATIII. The choice is made based on a comparison to the area under the normal CAT curve (thrombin potential) in its post-peak stage, equivalently, the differing ways that protein C and ATIII alter the CAT tail. If this normal area is already surpassed by the patient’s updated CAT estimate, then protein C is selected, otherwise it is ATIII. For all of the above modulations, coagulation factors are modulated only to the extent of their normal limits.

On the requisite four “co-” properties that an algorithm is typically scrutinized for, our proposed algorithm is convergent, complete, not complex, and correct. First, the GCM algorithm is guaranteed to converge to a set of personalized coagulation factor concentration recommendations, because we systematically manipulate an ordered list of a finite number of coagulation factors only once through the list. Next, our program is complete in the sense that if coagulation factor concentration values exist for all eight coagulation factors to generate a simulated CAT trajectory, then the algorithm will output one possible set. Consider that a set of coagulation factor concentrations always exists: this is the trivial set, consisting of the initial coagulation factor concentrations. Indeed, the algorithm presumes these concentrations at the start, before trying to modulate each concentration in turn. Earlier, we showed that our improved CAT prediction model can accurately predict CAT trajectories from coagulation factor concentrations, those that are measured before algorithm modulation. Thus, completeness is guaranteed. Third, our algorithm’s complexity is linear in the number of coagulation factors *n* (i.e., it is *O*(*n*) in big O notation); there is only one “for” loop in the pseudocode in Fig. 7, and we systematically examine each coagulation factor only once.

Finally, our GCM algorithm is correct, and we validated its outputs against clinical outcomes of CAT profile and normalized coagulation factor concentrations for eight trauma patients, dataset 6 in Supplementary Fig. 1 (demographic information in Supplementary Fig. 5a) who showed methodical recovery toward our normal goal. This eight-patient validation dataset (dataset 6) is different from, and is not a subset of, the 40 trauma patients (dataset 5) used for training the coagulation model (1) and for algorithm development. We validated the GCM algorithm for the first 24 h post hospital admission as this time period accounts for 80% of hemorrhage fatalities^15^. We selected intervention periods of 0, 6, 12, and 24 h for validation and comparison of our GCM algorithm to clinical data because trauma patient data in dataset 6 were collected at these time points.

**Fig. 9:**
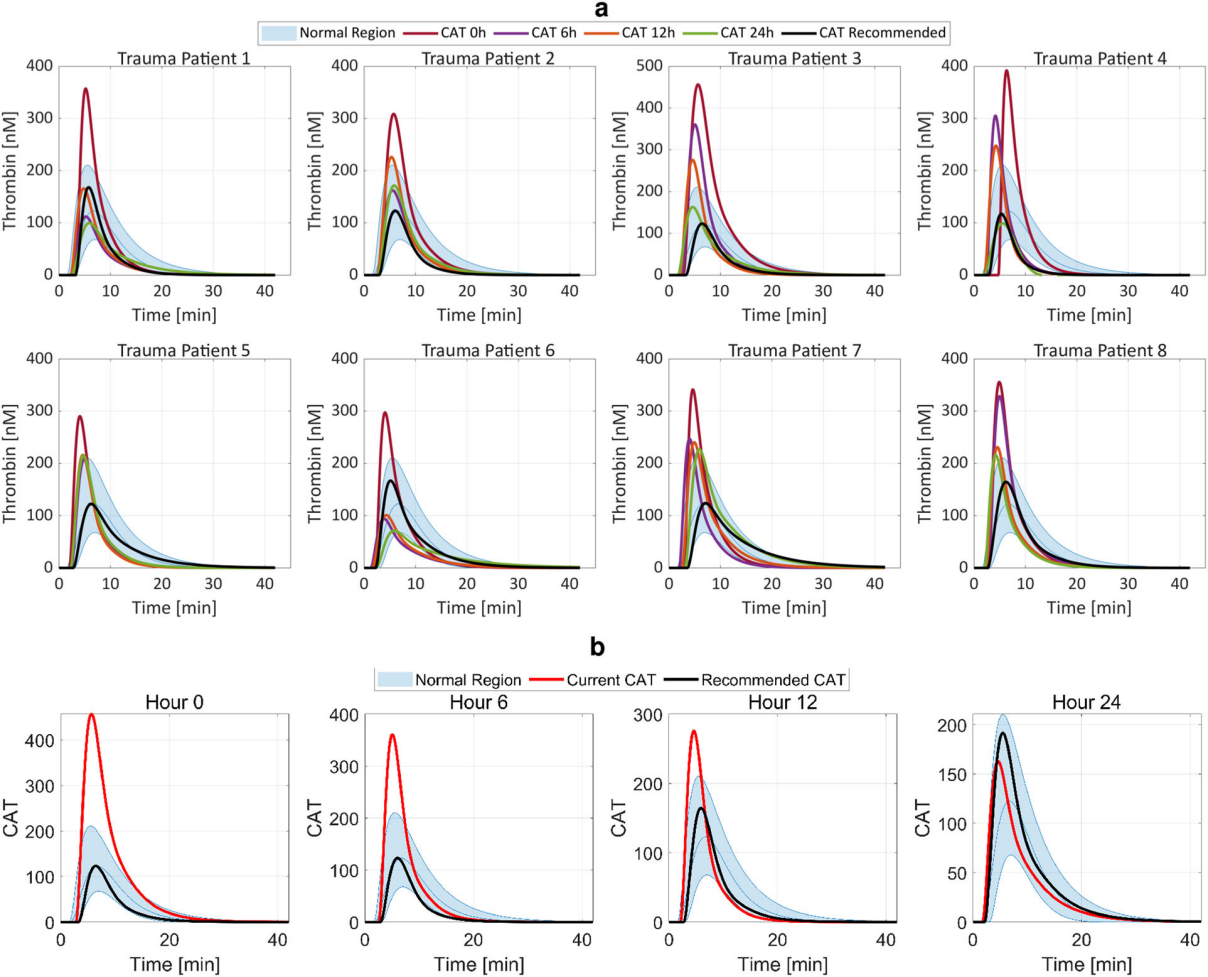
Our proposed GCM algorithm recommendations drive thrombin generation toward a normal region over time for eight trauma patient samples, validating the correctness and performance of the algorithm. **a** Estimated CAT trajectory from coagulation factor measurements for eight trauma patients over 24 h. The black line shows the GCM algorithm-recommended patient-specific CAT trajectory at 24 h if following the personalized coagulation factor recommendations for each patient. All recommended trajectories are visible inside normal ranges. Following the GCM algorithm recommendations shows desirable improvements over actual treatment received by eight trauma patients, in both CAT properties that are quantitatively compared to the normal region criteria (Supplementary Fig. 12a and b), and coagulation factor concentrations (Supplementary Fig. 12c and d). **b** Illustrative estimated CAT trajectory [nM] from coagulation factor measurements for Trauma Patient 3 at different instances over the first 24 h following hospital admission. The black line shows the GCM algorithm-recommended patient-specific CAT trajectory compared to the red line representing the actual CAT. This shows how the GCM algorithm dynamically adjusts the recommendations based on the most recent coagulation factor concentration measurements to move the CAT toward the normal region. In all instances, the recommended CAT is inside the normal region, leading the patient’s thrombin generation toward normal.

We contrasted CATs over 24 h, Fig. 9a, by estimating CAT trajectories from the coagulation factor concentrations in Supplementary Fig. 11. We illustrate the dynamic performance of the proposed GCM algorithm over 24 h for one of these trauma patients in Fig. 9b. Both panels show how the GCM algorithm recommendation adapts according to the most recent coagulation factor concentration measurements to guide the CAT toward the desired normal region. Comparing the recommended goal CAT to the normal region for three CAT properties of peak, peak-time, and area under the curve, the GCM algorithm’s recommendations show enhanced performance over the clinical practice that occurred, in mean and standard deviation percent error, for all properties over the first 24 h, Supplementary Fig. 12a, b. The program’s recommendations also rarely violate the normal CAT region. For the second goal to move coagulation factor concentrations to a normal range, none of the algorithm’s output coagulation factor concentrations violate the normal coagulation factor concentration range, in contrast to 38 violations that occurred during actual treatment of these eight patients at 24 h (Supplementary Fig. 12c), and numerous other violations that occurred at each of several intervening time points (Supplementary Fig. 12d).

## Discussion

The pressing need for trauma patient precision medicine treatments is well-documented, but the coagulopathy problem is complex, which restricts clinicians to using rules-of-thumb, generalized treatment protocols, and uncharacterized blood products. As a result, patient recovery often fluctuates between hypo-coagulable and hyper-coagulable states, with conditions that are complicated by the side effects from contemporary non-tailored approaches. This leads to high mortality and poor outcomes in even the best trauma centers. Goal-oriented, frequent, dynamic, and patient-specific interventions are believed to be the solution, especially if the administration of coagulation factors (blood proteins) can transfer a patient onto a desirable healing trajectory. However, no quantitative guidance exists on how to manipulate coagulation factor concentrations. Hence, to assist clinicians at the point-of-care, in this article we answer:

1. Is there merit to administering coagulation factors to treat trauma patients? We show significant evidence that the answer is yes, because patients who survive the first 24 h have coagulation factor concentrations that converge to values within normal ranges, and trauma patients who die in the first 24 h have coagulation factor concentrations that converge to values outside normal ranges.
2. Since there is merit to administering coagulation factors, how should these be administered to personalize trauma patient treatment? We provide a method and ordered sequence of operations for tuning coagulation factor concentrations, and develop the Goal-oriented Coagulation Management (GCM) algorithm as a fast, frequent, automated, and personalized treatment solution, which we have validated *in silico*. This algorithm systematically ensures satisfactory trauma patient coagulation recovery toward a goal by dynamically adjusting coagulation factors using patient-specifics indicated by an embedded clotting dynamics model. We identified a mechanistic progression of coagulation factor concentration changes to move a patient’s clotting state toward this goal. Our identified procedure recommends coagulation factor concentrations that are only within a predefined normal range.

To develop the GCM algorithm operations, we first needed a model that accurately captured patient-specific clotting dynamics. We improved upon a recent black-box model^48^ that was developed using system identification techniques, and that could predict the personalized thrombin dynamics of blood plasma samples from their quickly-measurable coagulation factor concentrations. We updated this model by incorporating an additional parameter to increase model flexibility without changing its structure, and we also added the effects of an eighth coagulation factor, protein C, because of its known coagulation importance in the literature. These two modifications substantially improved the model’s thrombin dynamics predictions, which were validated on data not used for model training.

We also verified *in silico* that administering coagulation factor concentrations changed the clotting that was described by this improved thrombin dynamics model. We noted saturating behavior for excessive coagulation factor concentration administration. This motivated our choice to keep coagulation factor levels between generally-accepted normal limits when modulated in a treatment scheme.

We validated GCM algorithm prediction performance *in silico* on data not used for training, by contrasting against metrics from actual trauma patients who recovered and also progressed toward normal. We showed that our method not only guides clotting predictions closer to normal, but does so while maintaining all coagulation factor concentrations within normal ranges, which was not the case in practice.

Our work offers a personalized control approach to trauma patient treatment, by updating a model of underlying clotting system dynamics, characterizing the effects and limitations of coagulation factor actuators, and then articulating a control algorithm to systematically achieve coagulation goals. We envision combining these advances in an automatic physical treatment device in future work, but our advances are also individually important. Our updated patient-specific clotting dynamics model can be leveraged in any personalized and dynamic treatment algorithm at the point-of-care, which itself can be repeated and iterated upon. The GCM algorithm is one such algorithm that facilitates the future automation of frequent, tailored clinical interventions in near real-time. An iterative approach permits quicker model updates, greater personalization, and a responsiveness to uncertainties, all of which will improve patient outcomes. Thus, the work in this article represents a considerable step toward frequent, personalized, and precision trauma patient resuscitation.

The next step is a large-scale in vitro experimental implementation of the GCM algorithm at shorter time points. Algorithm-recommended coagulation factor concentration increases in blood samples will be achieved by accurately adding specific recombinant coagulation factors, and algorithm-recommended coagulation factor concentration decreases will be achieved by accurately diluting samples or by augmenting inhibiting coagulation factors. We anticipate that this experimental implementation will necessitate a study in how to quantitatively optimize treatment administration. The results of our work are potentially relevant as a precision-medicine foundation for other coagulation disorders as well, such as hemophilia, von Willebrand disease, factor V Leiden, pulmonary embolism, deep vein thrombosis, stroke, and sickle cell disease.

## Methods

### Key resource table

A comprehensive table of all the reagents and resources that we used to conduct experiments, including for coagulation factor measurements and Calibrated Automated Thrombograms, are presented in Table 1. We also include the electronic resources that we used for simulations and analysis.

**Table 1:** Key resource table.

Reagent or resource	Source	Identifier
*Antibodies*		
STA-Asserachrom	Stago	Cat#00055
*Biological samples*		
STA-System Control N+P	Stago	Cat#00678
STA-Coag Control N+ABN	Stago	Cat#00679
STA-Liatest Control N+P	Stago	Cat#00526
STA-Unicalibrator	Stago	Cat#00675
STA-PTT Automate	Stago	Cat#00595
STA-C.K. Prest	Stago	Cat#00597
STA-Staclot Protein C	Stago	Cat#00737
STA-Stachrom ATIII	Stago	Cat#00671
STA-Neoplastine CI Plus	Stago	Cat#00376
STA-Deficient II	Stago	Cat#00740
STA-Deficient V	Stago	Cat#00744
STA-Deficient VII	Stago	Cat#00743
STA-Deficient VIII	Stago	Cat#00725
STA-Deficient IX	Stago	Cat#00724
STA-Deficient X	Stago	Cat#00738
Thrombin Calibrator	Stago	Cat#86192
PPP-Reagent	Stago	Cat#86193
*Chemicals, peptides, and recombinant proteins*		
STA-Owren Koller	Stago	Cat#00360
STA-Desorb U	Stago	Cat#00975
STA-CaCl2 0.025M	Stago	Cat#00367
STA-Cleaner Solution	Stago	Cat#00973
FluCa-Kit	Stago	Cat#86197
*Software and algorithms*		
MathWorks MATLAB	https://mathworks.com/	R2021a
GCM algorithm	This manuscript	https://github.com/SYBORGS-Lab/GCM-Algorithm
BioRender	https://biorender.com/	

### Coagulation factor concentration measurements

Coagulation factor concentrations were measured using the STA Compact Max^®^ as percent activity, which is with respect to the normal coagulation factor concentration in a healthy person. A normal range for coagulation factor concentrations is typically 60–140% activity^64,65^. Plasma samples were removed from −80 °C storage and thawed at room temperature. Reagents were prepared with DiH_2_O and left to stabilize for 30–60 min, as specified by the package insert. Owren-Koller diluent was used for patient samples, STA-Unicalibrator reagent was used to calibrate the system by measuring/defining ranges of new reagent lots (performed monthly), STA-System Control N+P and STA-Coag Control N+ABN were control reagents measured every 4 h and 8 h, respectively, and STA-Deficient reagent was used to measure the activity of a coagulation factor, e.g., STA-Deficient V was used for measuring factor V. The test automatically started after loading sample and reagents into the instrument. Given that quality control was repeated every 4 h, coagulation factor concentration measurements were performed once for each sample.

### Calibrated automated thrombogram

Plasma sample thrombin expression experimental data was obtained using the ThermoFisher Fluoroskan Microplate Fluorometer with Calibrated Automated Thrombogram software^58^, following the protocol in the software manual, explained briefly as follows. Plasma samples were removed from −80 °C storage and thawed at room temperature. Reagents were added to 96-well plates: thrombin calibrator reagent was used for the measurement control, and PPP-reagent was used to measure thrombin in normal or trauma samples. Plasma samples were added to plate wells, with three biological replicates, and the plate was loaded into a Fluoroskan Ascent platereader. Following a ten-minute incubation period at 37 °C, the test started automatically when the machine dispensed the FluCa reagent, which was pre-loaded. The generated thrombin was measured and recorded every 20 s. These measurements included three technical replicates.

### Model parameter fitting to experimental data

Model parameters of (1) were fit to experimental data using the MATLAB Simulink Design Optimization (SDO) toolbox. The input was defined as an impulse input with the desired magnitude, e.g., 5 pM of TF. The output to fit was the individual CAT profile experimental data. Solver tolerance was set to 1e−9. Starting from an initial parameter guess, the MATLAB SDO toolbox optimized parameter values of a transfer function model by minimizing the least square error between prediction and actual data using a trust region reflective algorithm. Following convergence, the finalized transfer function model parameters for each experimental sample were recorded and the poles computed.

**Fig. 10:**
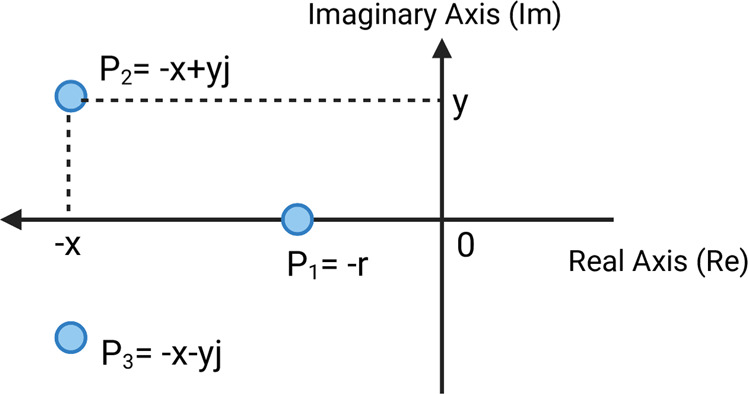
Dynamical system poles. Three pole orientation of coagulation model (1) in the complex plane.

Poles of a transfer function are the values for which the value of the denominator of the transfer function becomes zero. Therefore, to obtain the pole location values, we set the denominator of the fitted model equal to zero and solve the resultant equation, i.e., solve *s*^3^ + *K*_2_*s*^2^ + *K*_1_*s* + *K*_0_ = 0. Since this is a third-order system, the solution is a set of three numbers with real and imaginary parts, which can be plotted in the complex plane as in Fig. 10.

### Statistical analysis and significance test

For statistical analysis, we first performed Welch’s *t*-test for two unpaired samples (deceased, *x*, versus any one of the alive groups described in the main text, *y*) using (2).

2 $$t=\frac{{\mu }_{x}-{\mu }_{y}}{\sqrt{\frac{{S}_{x}^{2}}{n}-\frac{{S}_{y}^{2}}{m}}},$$

where *μ*_*x*_ and *μ*_*y*_ are the deceased and alive sample means, respectively; *S*_*x*_ and *S*_*y*_ are the sample standard deviations; and *n* and *m* are the sample sizes of *x* and *y*, respectively. Next, we calculated *p*-values for *α* = 0.05, i.e., 95% confidence interval, using MATLAB’s ttest2 function. We report the results by ns: not significant, *p* > 0.05; *p ≤ 0.05; **p ≤ 0.01; and ***p ≤ 0.001.

## Supplementary information

Supplementary Information

## Data Availability

This study was based on normal and trauma patient data arranged into nine datasets as shown in Supplementary Fig. 1. Normal data were obtained from a set of plasma samples from healthy individuals, with their CAT and coagulation factor concentration measurements characterized according to standard laboratory protocols as explained in the Methods section. Trauma patient data came from the Activation of Coagulation and Inflammation in Trauma study (ACIT), a previously described^63^ single-center prospective cohort study that followed severely-injured trauma patients from emergency department admission through discharge from hospitalization or death. Between February 2005 and May 2016, 1671 trauma patients (1367 male (81.45%), age 41.0 ± 18.6, ISS 17.7 ± 15.6) meeting criteria for highest triage activation level were enrolled into the study. Subsets of this dataset are used for various parts of this study, as indicated in the main text and in Supplementary Fig. 1. Patient demographics are in Supplementary Fig. 5. Exclusion criteria included patient age less than 15 years, pregnancy, incarceration, and transfer from outside hospital. Written consent was obtained from enrolled patients or their families or, rarely, in certain circumstances where these could not be obtained, a waiver of consent was utilized. The study was carried out with the approval of the University of California Institutional Review Board (reference number 10-04417). Data were collected at admission, 6, 12, and 24 h after injury. Upon request, the authors will share aggregate data that do not allow the identification of individuals, subject to a data sharing agreement.

## Code availability

The MATLAB controller GCM algorithm and underlying parameterized model is available at: https://github.com/SYBORGS-Lab/GCM-Algorithm. The algorithm code is available for personal use. However, this code is subject to the following commercial copyright: ^©^Copyright 2021 University of Florida Research Foundation, Inc. All commercial rights reserved.

## References

[1] Buchman TG (2016). Precision medicine for critical illness and injury. Crit. Care Med..

[2] Centers for Disease Control and Prevention. 10 leading causes of death, United States. https://wisqars-viz.cdc.gov:8006/lcd/home (2019).

[3] Stiell IG (2008). The OPALS major trauma study: impact of advanced life-support on survival and morbidity. CMAJ.

[4] Moore EE (2021). Trauma-induced coagulopathy. Nat. Rev. Dis. Primers.

[5] Peng N, Su L (2017). Progresses in understanding trauma-induced coagulopathy and the underlying mechanism. Chin. J. Traumatol..

[6] Maegele M, Paffrath T, Bouillon B (2011). Acute traumatic coagulopathy in severe injury: incidence, risk stratification, and treatment options. Deutsches Ärztebl. Int..

[7] Balvers K, Wirtz MR, van Dieren S, Goslings JC, Juffermans NP (2015). Risk factors for trauma-induced coagulopathy-and transfusion-associated multiple organ failure in severely injured trauma patients. Front. Med..

[8] Kudo D, Yoshida Y, Kushimoto S (2017). Permissive hypotension/hypotensive resuscitation and restricted/controlled resuscitation in patients with severe trauma. J. Intensive Care.

[9] Cohen, M. J. Coagulation perturbations after severe injury: translational approaches and the state of the science. In *Damage Control in Trauma Care*, 215–221 (Springer, 2018).

[10] Bradley, M. et al. Prediction of venous thromboembolism using clinical and serum biomarker data from a military cohort of trauma patients. *BMJ Mil Health*10.1136/bmjmilitary-2019-001393 (2020). 10.1136/bmjmilitary-2019-001393 32139417

[11] Holcomb JB (2015). Transfusion of plasma, platelets, and red blood cells in a 1: 1: 1 vs a 1: 1: 2 ratio and mortality in patients with severe trauma: the PROPPR randomized clinical trial. JAMA.

[12] Marsden M (2018). Outcomes following trauma laparotomy for hypotensive trauma patients: a UK military and civilian perspective. J. Trauma Acute Care Surg..

[13] Harvin JA (2017). Mortality following emergent trauma laparotomy: a multicenter, retrospective study: mortality after emergent trauma laparotomy. J. Trauma Acute Care Surg..

[14] Acosta JA (1998). Lethal injuries and time to death in a level I trauma center. J. Am. College Surg..

[15] Tisherman SA (2015). Detailed description of all deaths in both the shock and traumatic brain injury hypertonic saline trials of the Resuscitation Outcomes Consortium. Ann. Surg..

[16] Cotton, B. A. et al. Damage control resuscitation is associated with a reduction in resuscitation volumes and improvement in survival in 390 damage control laparotomy patients. *Ann. Surg.* **254**, 598–605 (2011). 10.1097/SLA.0b013e318230089e PMC3816774 21918426

[17] Holcomb JB (2013). The prospective, observational, multicenter, major trauma transfusion (PROMMTT) study: comparative effectiveness of a time-varying treatment with competing risks. JAMA Surg..

[18] Langan NR, Eckert M, Martin MJ (2014). Changing patterns of in-hospital deaths following implementation of damage control resuscitation practices in US forward military treatment facilities. JAMA Surg..

[19] Roquet F (2019). Association of early, high plasma-to-red blood cell transfusion ratio with mortality in adults with severe bleeding after trauma. JAMA Network Open.

[20] Scalea TM (2008). Early aggressive use of fresh frozen plasma does not improve outcome in critically injured trauma patients. Ann. Surg..

[21] Cotton BA (2012). Hyperfibrinolysis at admission is an uncommon but highly lethal event associated with shock and prehospital fluid administration. J. Trauma Acute Care Surg..

[22] Mesar T (2017). Association between ratio of fresh frozen plasma to red blood cells during massive transfusion and survival among patients without traumatic injury. JAMA Surg..

[23] Oliveros Rodríguez H (2020). Mortality in civilian trauma patients and massive blood transfusion treated with high vs low plasma: red blood cell ratio. Systematic review and meta-analysis. Revista Colombiana de Anestesiología.

[24] Johnson JL (2010). Effect of blood products transfusion on the development of postinjury multiple organ failure. Arch. Surg..

[25] Cantle PM, Cotton BA (2017). Prediction of massive transfusion in trauma. Critical Care Clin..

[26] Černý, V. et al. Variations and obstacles in the use of coagulation factor concentrates for major trauma bleeding across Europe: outcomes from a European expert meeting. *Eur. J. Trauma Emerg. Surg.*10.1007/s00068-020-01563-2 (2021). 10.1007/s00068-020-01563-2 PMC7782571 33399876

[27] Giangrande P (2017). Clinical evaluation of glycoPEGylated recombinant FVIII: efficacy and safety in severe haemophilia A. Thromb. Haemost..

[28] Giangrande P (2017). Kreuth IV: European consensus proposals for treatment of haemophilia with coagulation factor concentrates. Haemophilia.

[29] Innerhofer P (2017). Reversal of trauma-induced coagulopathy using first-line coagulation factor concentrates or fresh frozen plasma (RETIC): a single-centre, parallel-group, open-label, randomised trial. Lancet Haematol..

[30] Godier A, Greinacher A, Faraoni D, Levy J, Samama C (2018). Use of factor concentrates for the management of perioperative bleeding: guidance from the SSC of the ISTH. J. Thromb. Haemost..

[31] Gratz, J. et al. Comparison of fresh frozen plasma vs. coagulation factor concentrates for reconstitution of blood: An in vitro study. *Eur. J. Anaesthesiol.* **37**, 879–888 (2020). 10.1097/EJA.0000000000001202 32251150

[32] Dutton RP (2004). Factor VIIa for correction of traumatic coagulopathy. J. Trauma Acute Care Surg..

[33] Joseph B (2012). Factor IX complex for the correction of traumatic coagulopathy. J. Trauma Acute Care Surg..

[34] Turpie AG (2007). Oral, direct factor Xa inhibitors in development for the prevention and treatment of thromboembolic diseases. Arterioscler. Thromb. Vasc. Biol..

[35] Cohen MJ (2012). Critical role of activated protein C in early coagulopathy and later organ failure, infection and death in trauma patients. Ann. Surg..

[36] Simmons J, Powell M (2016). Acute traumatic coagulopathy: pathophysiology and resuscitation. Br. J. Anaesth..

[37] Schöchl H, Schlimp CJ (2014). Trauma bleeding management: the concept of goal-directed primary care. Anesth. Analg..

[38] Maegele M, Nardi G, Schöchl H (2017). Hemotherapy algorithm for the management of trauma-induced coagulopathy: the German and European perspective. Curr. Opin. Anesthesiol..

[39] Juffermans NP (2019). Towards patient-specific management of trauma hemorrhage: the effect of resuscitation therapy on parameters of thromboelastometry. J. Thromb. Haemost..

[40] Stein AL (2019). Impact of a goal-directed factor-based coagulation management on thromboembolic events following major trauma. Scand. J. Trauma, Resusc. Emerg. Med..

[41] Selby R (2009). Hypercoagulability after trauma: hemostatic changes and relationship to venous thromboembolism. Thromb. Res..

[42] Haider AH (2013). Disparities in trauma care and outcomes in the United States: a systematic review and meta-analysis. J. Trauma Acute Care Surg..

[43] Liu NT, Salinas J (2017). Machine learning for predicting outcomes in trauma. Shock.

[44] Christie SA, Conroy AS, Callcut RA, Hubbard AE, Cohen MJ (2019). Dynamic multi-outcome prediction after injury: Applying adaptive machine learning for precision medicine in trauma. PLoS ONE.

[45] Pressly MA, Parker RS, Neal MD, Sperry JL, Clermont G (2020). Accelerating availability of clinically-relevant parameter estimates from thromboelastogram point-of-care device. J. Trauma Acute Care Surg..

[46] Duque P, Mora L, Levy JH, Schöchl H (2020). Pathophysiological response to trauma-induced coagulopathy: a comprehensive review. Anesth. Analg..

[47] Gonzalez, E., Moore, H. B. & Moore, E. E. *Trauma Induced Coagulopathy* (Springer, 2016).

[48] Menezes, A. A., Vilardi, R. F., Arkin, A. P. & Cohen, M. J. Targeted clinical control of trauma patient coagulation through a thrombin dynamics model. *Sci. Transl. Med.* **9**, eaaf5045 (2017). 10.1126/scitranslmed.aaf5045 28053156

[49] Hockin MF, Jones KC, Everse SJ, Mann KG (2002). A model for the stoichiometric regulation of blood coagulation. J. Biol. Chem..

[50] Mann KG (2012). Is there value in kinetic modeling of thrombin generation? Yes. J. Thromb. Haemost..

[51] Stein, P., Kaserer, A., Spahn, G. H. & Spahn, D. R. Point-of-care coagulation monitoring in trauma patients. In *Seminars in Thrombosis and Hemostasis*, Vol. 43, 367–374 (Thieme Medical Publishers, 2017). 10.1055/s-0037-1598062 28297730

[52] Adams, R. L. & Bird, R. J. Coagulation cascade and therapeutics update: relevance to nephrology. Part 1: overview of coagulation, thrombophilias and history of anticoagulants. *Nephrology* **14**, 462–470 (2009). 10.1111/j.1440-1797.2009.01128.x 19674315

[53] Cardenas, J. C. et al. Measuring thrombin generation as a tool for predicting hemostatic potential and transfusion requirements following trauma. *J. Trauma Acute Care Surg*. **77**, 839–845 (2014). 10.1097/TA.0000000000000348 25099452

[54] Walsh M (2016). Targeted thromboelastographic (TEG) blood component and pharmacologic hemostatic therapy in traumatic and acquired coagulopathy. Curr. Drug Targets.

[55] Narayanan S (1999). Multifunctional roles of thrombin. Ann. Clin. Lab. Sci..

[56] Senst, B., Tadi, P., Basit, H. & Jan, A. Hypercoagulability. In *StatPearls* (StatPearls Publishing, 2020). 30855839

[57] Brohi K (2007). Acute traumatic coagulopathy: initiated by hypoperfusion: modulated through the protein C pathway?. Ann. Surg..

[58] Hemker H (2002). The calibrated automated thrombogram (CAT): a universal routine test for hyper-and hypocoagulability. Pathophysiol. Haemost. Thromb..

[59] Gonzalez E (2006). Early coagulopathy and massive transfusion (MT) in civilian trauma and combat casualties. Shock.

[60] Luan D, Szlam F, Tanaka KA, Barie PS, Varner JD (2010). Ensembles of uncertain mathematical models can identify network response to therapeutic interventions. Mol. BioSyst..

[61] Brummel-Ziedins KE (2012). The prothrombotic phenotypes in familial protein C deficiency are differentiated by computational modeling of thrombin generation. PLoS ONE.

[62] Gupta S (2019). Mathematical model of thrombin generation and bleeding phenotype in Amish carriers of Factor IX: C deficiency vs. controls. Thromb. Res..

[63] Cohen MJ (2009). Protein C depletion early after trauma increases the risk of ventilator-associated pneumonia. J. Trauma Acute Care Surg..

[64] Wakeman, L. et al. N*ew Coagulation Assays Reference Ranges for Healthy Adults Using the Modern Sysmex CA-1500 Coagulometer* (American Society of Hematology, 2005).

[65] Key, N. S., Makris, M. & Lillicrap, D. *P**ractical Hemostasis and Thrombosis* (John Wiley & Sons, 2017).

[66] Dorf, R. C. & Bishop, R. H. *Modern Control Systems*, 12th edn. (Pearson Prentice Hall, 2011).

[67] Mallat SG, Zhang Z (1993). Matching pursuits with time-frequency dictionaries. IEEE Trans. Signal Process..

[68] Walker FJ, Fay PJ (1992). Regulation of blood coagulation by the protein C system. FASEB J..

[69] Kutcher, M. E. et al. Extracellular histone release in response to traumatic injury: implications for a compensatory role of activated protein C. *J. Trauma Acute Care Surg.* **73**, 1389–1394 (2012). 10.1097/TA.0b013e318270d595 PMC3577065 23188230

[70] James, G., Witten, D., Hastie, T. & Tibshirani, R. *An Introduction to Statistical Learning* (Springer, 2013).

[71] Pintelon, R. & Schoukens, J. *System Identification: A Frequency Domain Approach* (John Wiley & Sons, 2012).

[72] Sakamoto, Y., Ishiguro, M. & Kitagawa, G. *Akaike Information Criterion Statistics* (Dordrecht Reidel, 1986).

