# Supplementary material for: Personalized modulation of coagulation factors using a thrombin dynamics model to treat trauma-induced coagulopathy
Source: NPJ Syst Biol Appl. 2021 Dec 7;7:44. doi: 10.1038/s41540-021-00202-9 (PMC8651743; doi:10.1038/s41540-021-00202-9)
Supplement: Supplementary file 1 — Supplementary Information [file 41540_2021_202_MOESM1_ESM.pdf]

Personalized modulation of coagulation factors using a thrombin dynamics model to treat trauma-induced coagulopathy

Damon E. Ghetmiri[1], Mitchell J. Cohen[2], Amor A. Menezes[1,3,4,*]

[1] Department of Mechanical and Aerospace Engineering, University of Florida, Gainesville, FL, USA

[2] Department of Surgery, University of Colorado Anschutz Medical Campus, Aurora, CO, USA

[3] J. Crayton Pruitt Family Department of Biomedical Engineering, University of Florida, Gainesville, FL, USA

[4] Department of Agricultural and Biological Engineering, University of Florida, Gainesville, FL, USA

[*] Corresponding author: amormenezes@ufl.edu

Supplementary Materials

List of Figures

1 Dataset overview and summary . . . 3

2 Static machine learning on the coagulation factor concentrations of 1,671 trauma patients is uninformative for patient measures . . . 4

3 Static machine learning on the coagulation factor concentrations of 1,671 trauma patients cannot classify by injury severity . . . 5

4 Static machine learning clustering on 39 variables of 1,671 trauma patients cannot accurately classify mortality . . . 6

5 Demographic information of trauma patients who survived 24 h and who had a complete set of measurements . . . 7

6 Trauma patient coagulation factor concentration time history over the first 24 h lacks correlation with mortality, transfusion, or thromboembolic events . . . 8

7 Time history of the change in trauma patient coagulation factor concentrations lacks correlation with the number of FFP units received . . . 9

8 Identifying a normal CAT region for the GCM algorithm to drive patient thrombin generation to . 10

9 Validation of the identified normal region . . . 11

10 Effects of coagulation factor concentration changes on one trauma patient's estimated CAT that are identified in real-time as mapping functions to enable GCM algorithm recommendations . . . 12

11 The change in coagulation factor concentrations of eight trauma patients who showed methodical recovery toward normal over 24 h does not follow a clear pattern . . . 13

12 A comparison between actual and GCM algorithm-recommended recovery using CAT criteria and coagulation factor concentrations in eight trauma patients shows the advantages of the proposed algorithm 14

List of Tables

1 Mean relative error of five validation samples at each time point compared to the identified normal thrombin region lower-bound and upper-bound 11

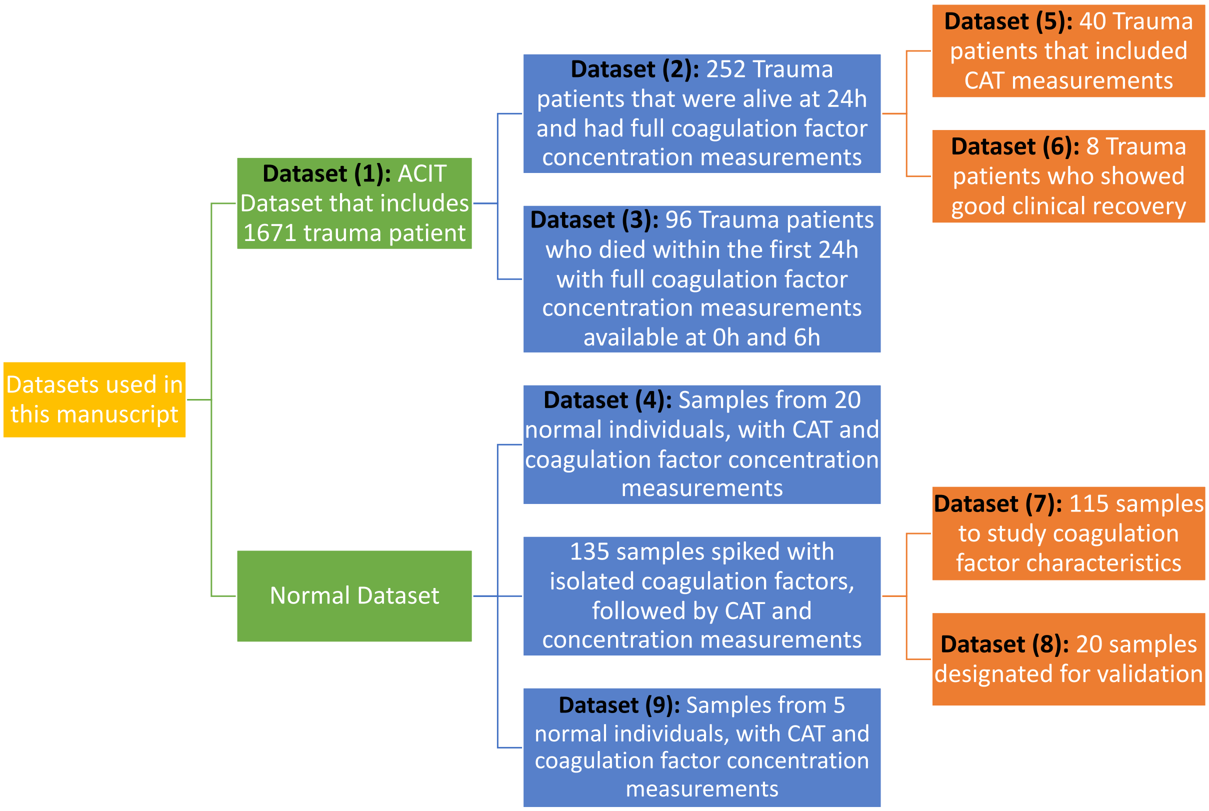

Objective	Content	Datasets
Static machine learning	Supplementary Figs. 2, 3, and 4	(1)
Time history of coagulation factor concentrations	Fig. 4, and Supplementary Figs. 6 and 7	(1)
Heatmap of coagulation factor concentration dynamics	Fig. 3a	(2)
Coagulation factor concentration dynamics in dead and alive patients (p-value)	Fig. 3b	(2) and (3)
Coagulation model: Training	Fig. 5a,b,c	(4) and (5)
Coagulation model: Validation, five-fold cross validation	Fig. 5d	(4) and (5)
Coagulation model: Validation on data not used for training	Fig. 5e	(8)
Characterization of unique effects of coagulation factors as actuators	Fig. 6	(4),(5), and (7)
Coagulation factor actuator effect: Validation on data not used for characterization	Fig. 5e	(8)
GCM Algorithm development	Figs. 7 and 8	(4) and (5)
GCM Algorithm comparison to clinical interventions	Fig. 9	(6)

Supplementary Fig. 1: Dataset overview and summary. Datasets used for different results in this article, including model development, analysis, and simulation. As indicated, separate datasets were used for training and validation.

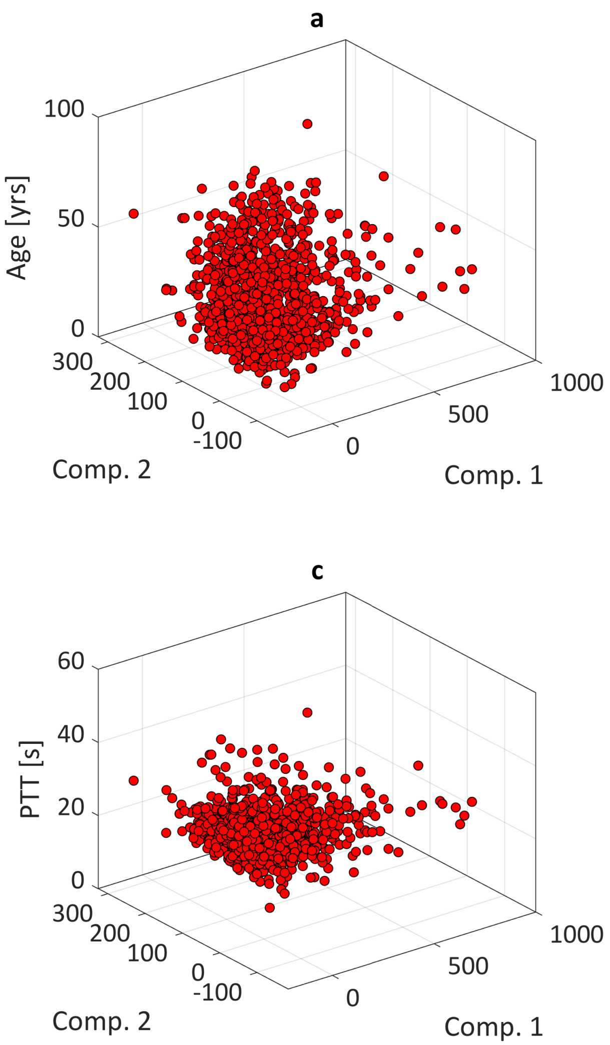

Supplementary Fig. 2: Static machine learning on the coagulation factor concentrations of 1,671 trauma patients (dataset 1) is uninformative for patient measures. A principal component analysis (PCA) on the coagulation factor concentrations of 1,671 trauma patients shows no correlation between the first two principal components and patient measures like **(a)** age, **(b)** injury severity score ISS, **(c)** partial thromboplastin time PTT, or **(d)** international normalized ratio INR.

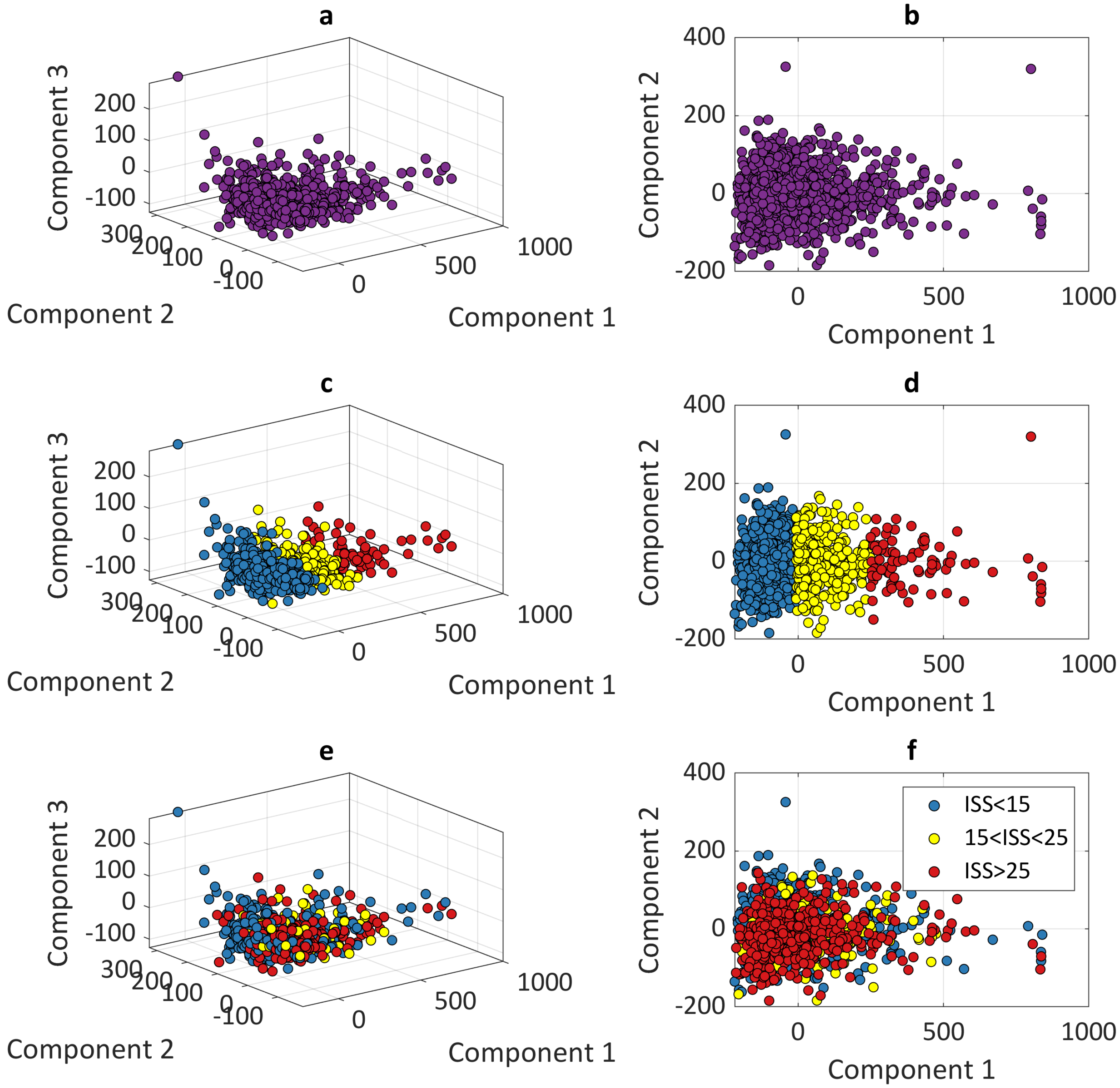

Supplementary Fig. 3: Static machine learning on the coagulation factor concentrations of 1,671 trauma patients (dataset 1) cannot classify by injury severity. (a) The first three principal components from a PCA on the coagulation factor concentrations of 1,671 trauma patients, and **(b)** their projection into the plane of the first two components. **(c)** and **(d)** K-means clustering into three clusters applied to (a) and (b), respectively. **(e)** Clustering according to ISS shows substantial overlap between classes, and a mismatch to the k-means PCA clusters (blue: ISS < 16, yellow: $15 <$ ISS < 25, red: ISS > 25) in both 3D (c) and **(f)** 2D (d) planes.

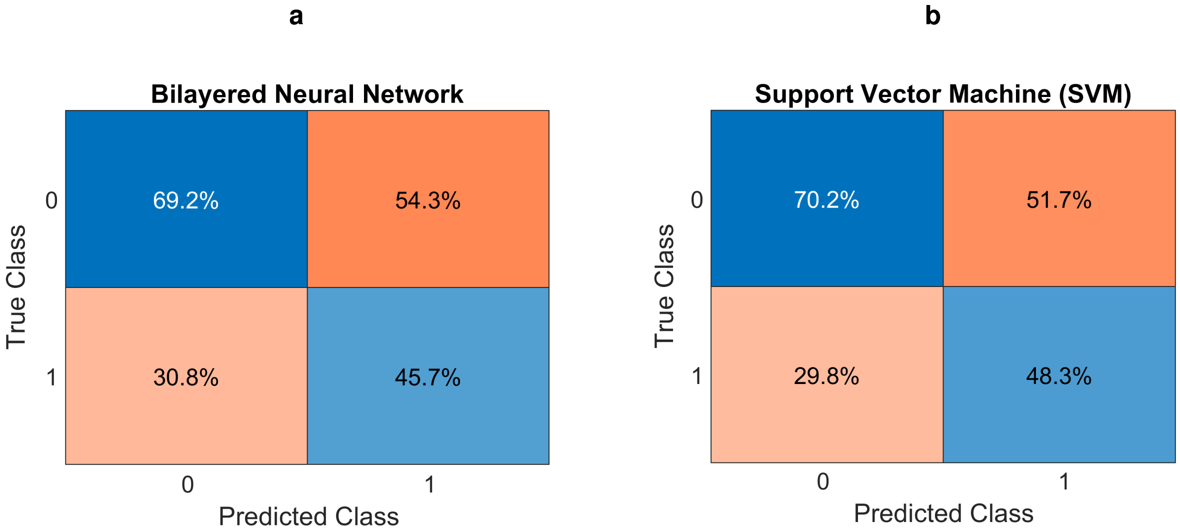

Supplementary Fig. 4: Static machine learning clustering on 39 variables (including coagulation factor concentrations, vital signs, and patient demographics) of 1,671 trauma patients (dataset 1) cannot accurately classify mortality. Dataset 1 was randomly split 75% for training and 25% for validation. **(a)** A bilayered neural network with 10 nodes in each layer has a mean mortality prediction accuracy of 60.7% on the validation dataset. **(b)** A support vector machine marginally improves mean mortality prediction accuracy to 62.6% on the validation dataset. Both methods have a greater than 50% incorrect prediction of survival. Figure axes use 0 for dead and 1 for alive.

a

Characteristic	Mean ± std. dev. or percentage (no. out of 252)
Age	43.4 ± 18.7
Male/female	79.4% (200) / 20.6% (52)
ISS	26.9 ± 14.7
Blunt	71.4% (180)
TBI present/not present	55.5% (140) / 44.5% (112)
Alive/dead at 28 days	83.3% (215) / 14.7% (37)
Alive/dead	82.9% (209) / 17.1% (43)
PTT (s) at admission	29.5 ± 7.7
PT (s) at admission	14.8 ± 2.4
INR at admission	1.2 ± 0.3

b

Characteristic	Mean ± std. dev. or percentage (no. out of 8)
Age	47.6 ± 13.5
Male/female	62.5% (5) / 37.5% (3)
ISS	31.0 ± 17.3
Blunt	50% (4)
TBI present/not present	37.5% (3) / 62.5% (5)
Alive/dead at 28 days	100% (8) / 0% (0)
Alive/dead	100% (8) / 0% (0)
PTT (s) at admission	29.1 ± 7.0
PT (s) at admission	14.4 ± 1.1
INR at admission	1.2 ± 0.1

Supplementary Fig. 5: Demographic information of trauma patients in dataset 1 who survived 24 h and who had a complete set of measurements, dataset 2. (a) All 252 trauma patients in our coagulation factor concentration analysis. This patient group averaged a high ISS and a high percentage of blunt injury. **(b)** The eight trauma patients used to validate the treatment algorithm in this article, dataset 6. This patient group averaged a high ISS and a high percentage of blunt injury. These patients successfully recovered and were discharged from hospital.

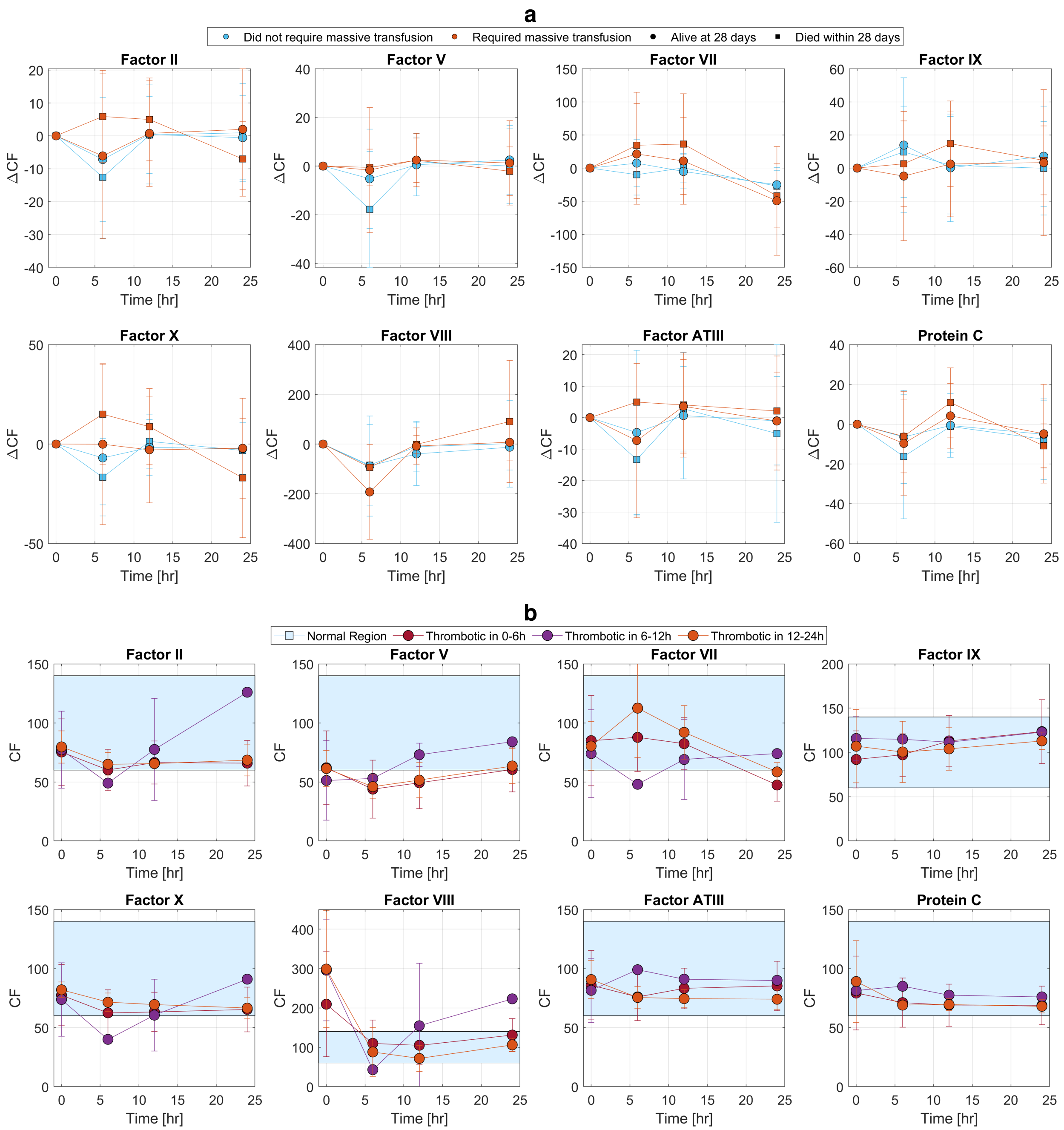

Supplementary Fig. 6: Trauma patient coagulation factor concentration time history over the first 24 h lacks correlation with mortality, transfusion, or thromboembolic events. (a) Mean ± one standard deviation of the changes in coagulation factor concentrations (ΔCF) during the first 24 h after hospital admission, for factors II, V, VII, VIII, IX, X, ATIII, and protein C of 252 trauma patients, dataset 2 (demographics in Supplementary Fig. 5 (a)), grouped by their need for massive transfusion and their mortality at 28 days. No trends are discernible, predominantly due to a lack of characterization of FFP units administered to each patient. **(b)** Mean ± one standard deviation of the CFs of 19 of the 252 trauma patients that experienced a thrombotic event during the first 24 h after hospital admission, grouped by event time window. Individual coagulation factor concentrations do not inform of whether a patient is at risk of a thrombotic event in different time windows, compared to other groups. Moreover, these patients experienced a thrombotic event despite the mean of most CFs being normal or near-normal, where normal is the coagulation factor concentration range of 60–140% activity. Units of coagulation factor concentrations are reported as percent activity.

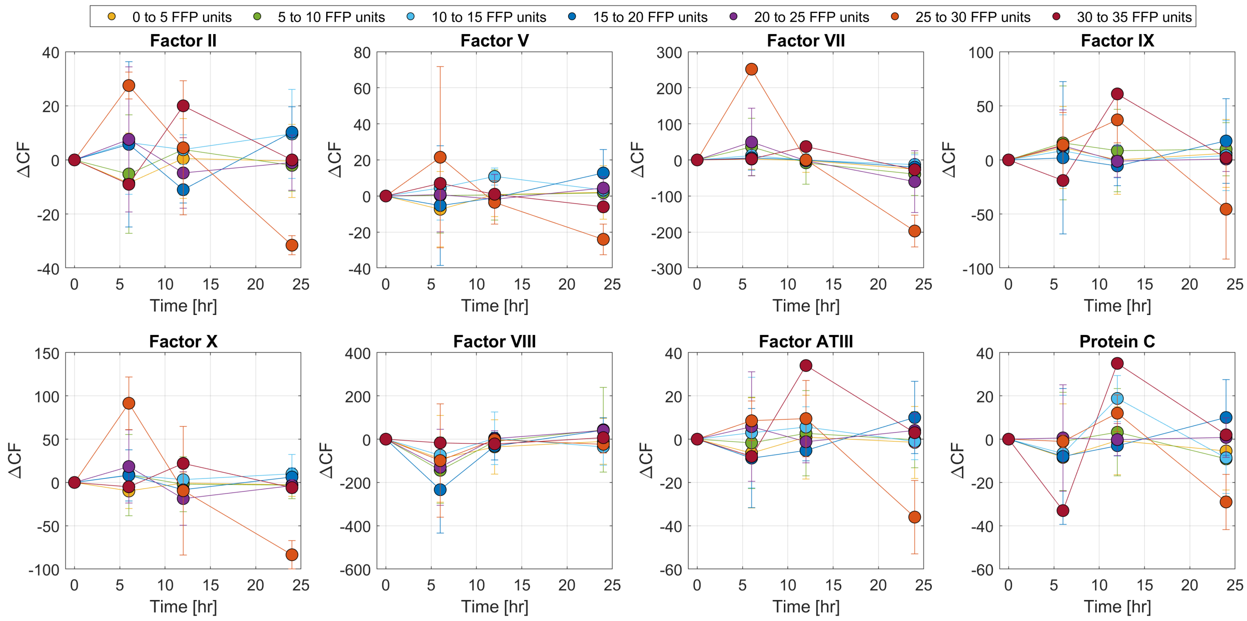

Supplementary Fig. 7: Time history of the change in trauma patient coagulation factor concentrations lacks correlation with the number of FFP units received. Mean ± one standard deviation of the changes in coagulation factor concentrations (ΔCF) during the first 24 h after hospital admission, for factors II, V, VII, VIII, IX, X, ATIII, and protein C of 252 trauma patients, dataset 2 (demographics in Supplementary Fig. 5 (a)), grouped by the number of FFP units received. These changes are inconsistent, which is attributable to the lack of characterization of administered plasma and the inherent variability in each FFP unit. Units of coagulation factor concentrations are reported as percent activity.

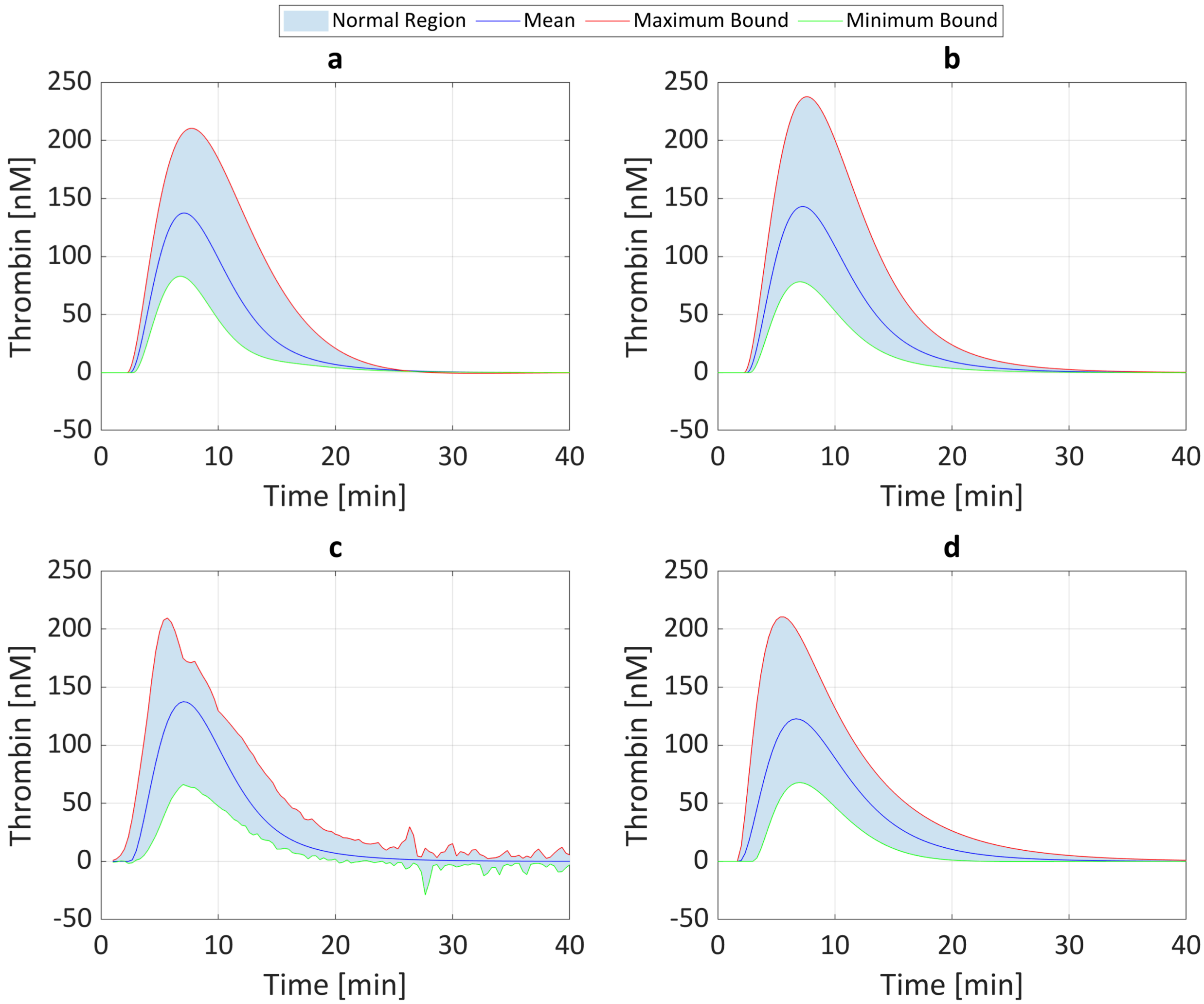

Supplementary Fig. 8: Identifying a normal CAT region for the GCM algorithm to drive patient thrombin generation to. Four candidate normal CAT regions are possible using experimental CAT data from 20 normal donor samples, dataset 4. (a) Mean $\pm$ 0.5 standard deviation of fitted model (1) parameters. **(b)** Mean $\pm$ 0.5 standard deviation of poles from the fitted model (1). **(c)** Mean, minimum, and maximum of all experimental data at each time point. **(d)** Fitted model to the data of panel (c). Panel (d) is chosen as the goal normal CAT region, since it offers the best representation of the data, and is the most restrictive.

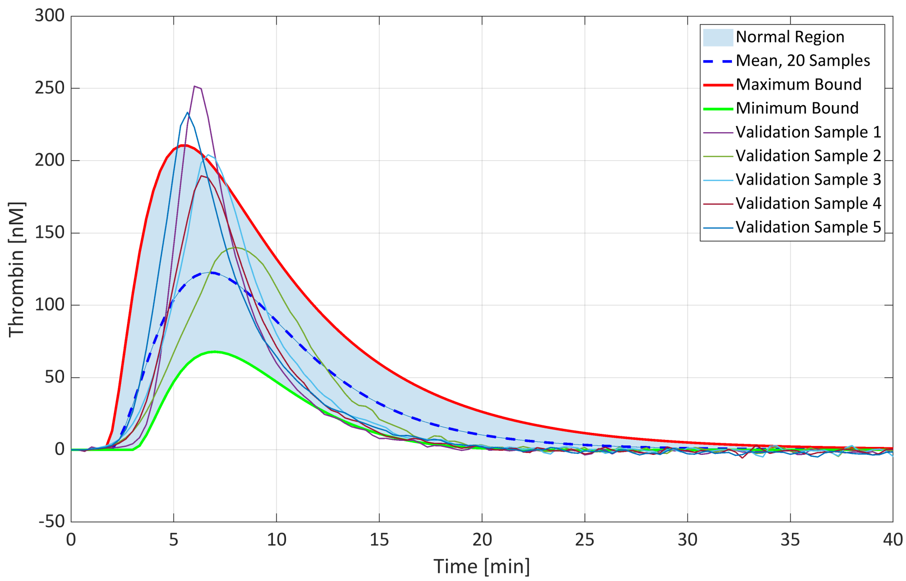

Supplementary Fig. 9: Validation of the identified normal region. We validate how well this thrombin region represents normal by comparing it to five validation samples that were not used to identify this region.

Supplementary Table 1: Mean relative error of five validation samples at each time point compared to the identified normal thrombin region lower-bound and upper-bound

MRE	Sample 1	Sample 2	Sample 3	Sample 4	Sample 5
Minimum bound	8.398%	0.0%	0.0%	4.448%	0.0%
Maximum bound	4.874%	0.0%	2.484%	0.0%	3.620%
Mean of Min and Max MRE	6.636%	0.0%	1.242%	2.224%	1.810%

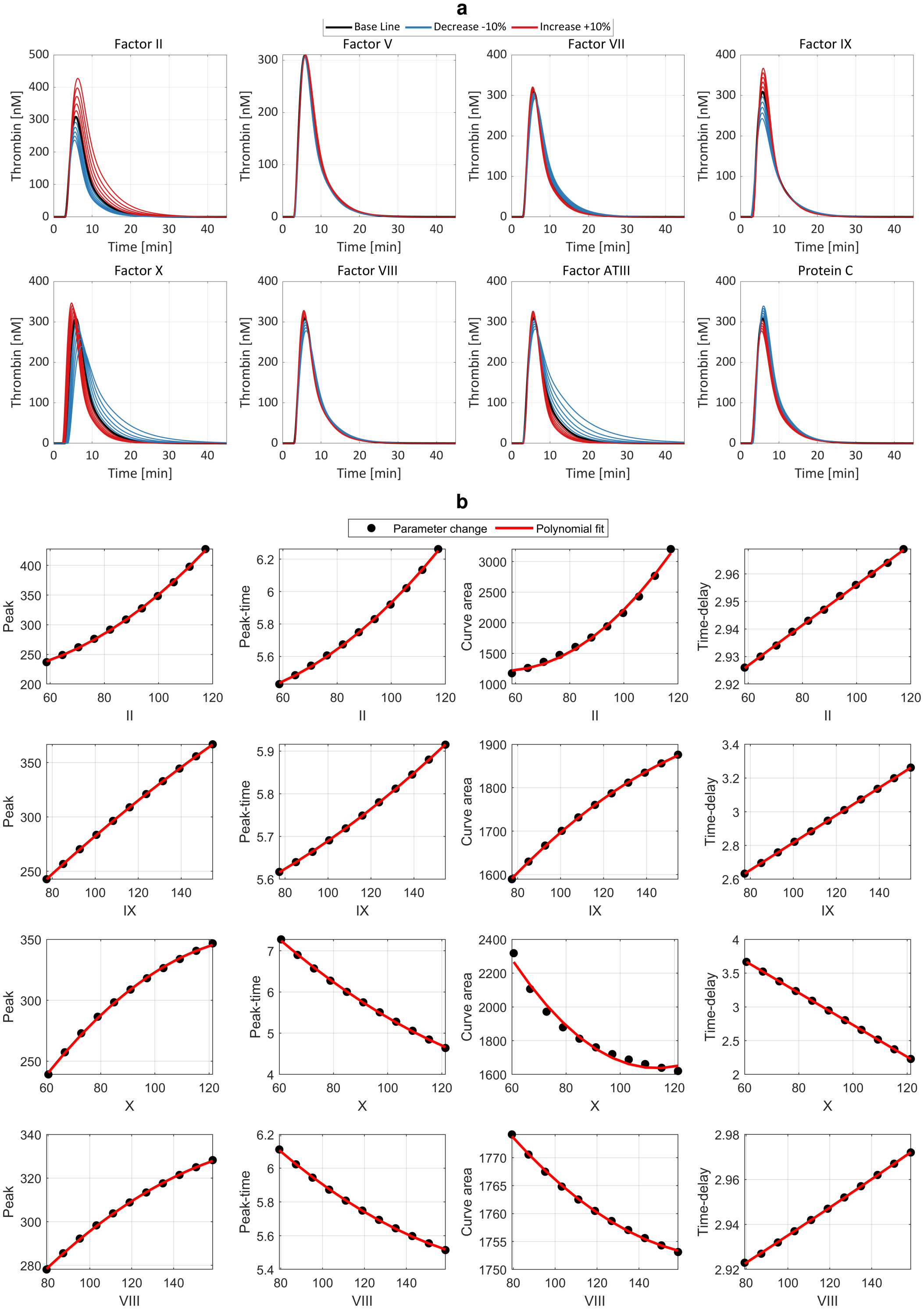

Supplementary Fig. 10: Effects of coagulation factor concentration changes on one trauma patient's estimated CAT that are identified in real-time as mapping functions to enable GCM algorithm recommendations. (a) CAT changes from -50% to +50% of initial coagulation factor concentrations, in 10% increments. **(b)** Quadratic polynomial-based patient-specific mappings from coagulation factor concentration changes to estimated CAT properties have excellent fits (mean $R^2 = 0.9996$), enabling property manipulation as desired.

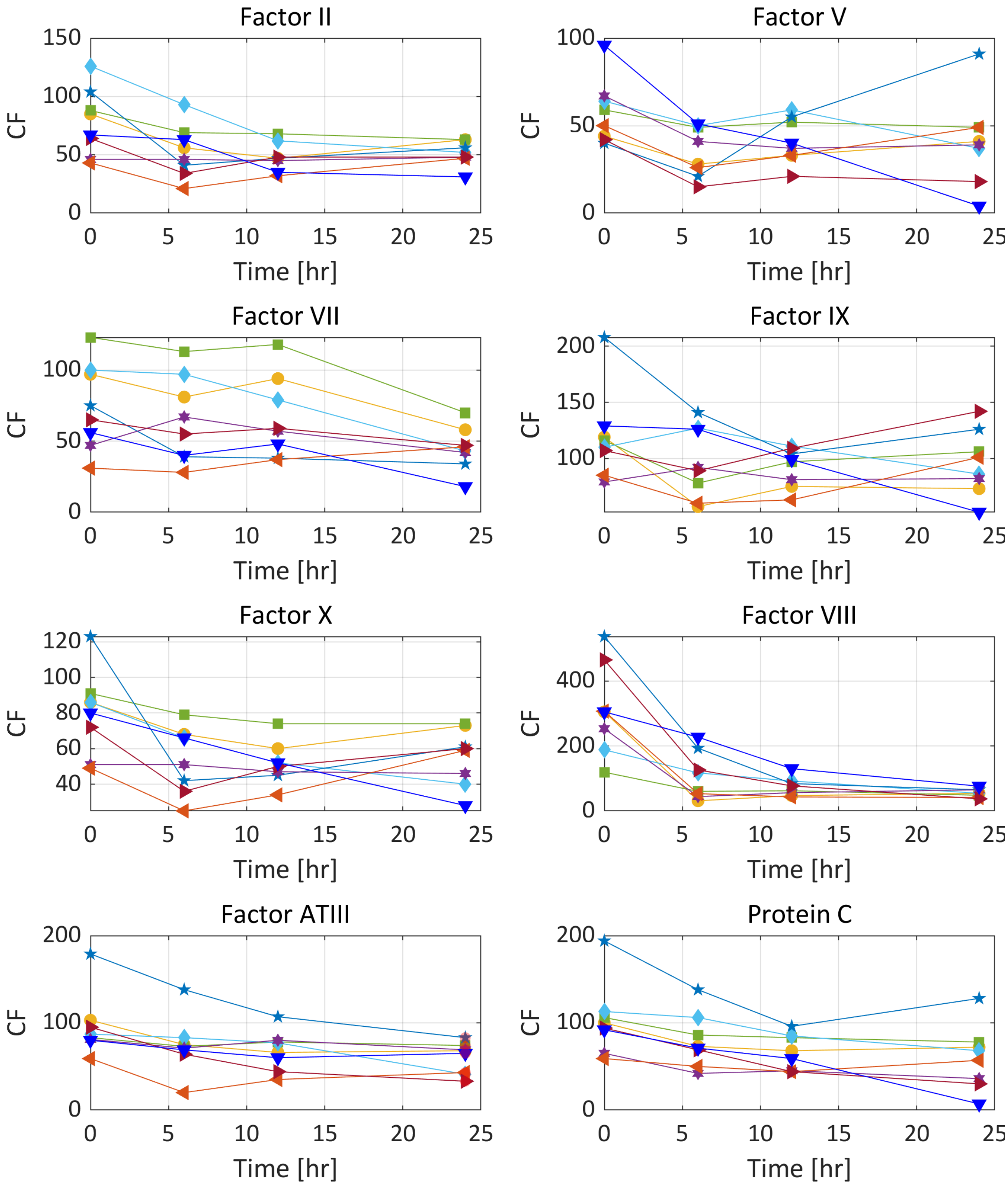

Supplementary Fig. 11: The change in coagulation factor concentrations of eight trauma patients who showed methodical recovery toward normal over 24 h does not follow a clear pattern. Patient demographic information is in Supplementary Fig. 5b. No pattern in coagulation factor concentration changes is apparent, necessitating the personalized treatment that is afforded by the GCM algorithm in this article. Units of coagulation factor concentrations are reported as percent activity.

a

Inside normal range | Outside normal range

Trauma sample	Peak error (%)		Peak-time error (%)		Curve area error (%)	
	Actual at 24 h	Recom.	Actual at 24 h	Recom.	Actual at 24 h	Recom.
1	18.98	36.97	13.61	15.07	25.09	21.17
2	39.76	0.38	13.61	12.14	2.48	36.36
3	32.85	0.35	32.64	6.28	2.75	30.91
4	19.33	4.55	18.00	22.39	54.08	52.27
5	75.47	0.16	29.71	9.21	1.22	4.03
6	41.77	35.92	13.67	25.32	30.56	7.11
7	85.21	0.94	13.62	3.96	52.80	12.06
8	75.87	34.04	38.50	9.21	6.69	2.50
**Mean ± Std. dev.**	**48.65 ± 26.49**	**14.17 ± 17.86**	**21.66 ± 10.29**	**12.95 ± 7.56**	**21.90 ± 22.35**	**20.80 ± 17.79**

b

CAT	Actual at 0 h	Actual at 6 h	Actual at 12 h	Actual at 24 h	Recom.
Violations	18	12	14	6	2

c

Inside normal range | Outside normal range

Trauma sample	24 hours								Recommendation							
	II	V	VII	IX	X	VIII	ATIII	PC	II	V	VII	IX	X	VIII	ATIII	PC
1	63	41	58	73	73	54	68	72	60	60	97	60	109	140	103	100
2	63	49	70	106	74	48	74	78	75	60	123	60	105	82	105	106
3	52	37	43	86	40	54	41	68	74	64	100	60	122	140	110	113
4	56	91	34	126	61	65	83	128	60	60	75	60	116	140	130	194
5	48	39	42	82	46	65	69	36	66	67	60	60	90	133	81	65
6	47	49	46	101	59	41	43	57	60	60	60	87	72	140	61	109
7	48	18	47	142	60	37	33	30	82	60	65	60	122	140	95	94
8	31	4	18	52	28	76	65	7	60	96	60	109	89	140	80	92

d

Factor Concentration	Actual at 0 h	Actual at 6 h	Actual at 12 h	Actual at 24 h	Recom.
Violations	24	31	34	38	0

Supplementary Fig. 12: A comparison between actual and GCM algorithm-recommended recovery using CAT criteria and coagulation factor concentrations in eight trauma patients, dataset 6, shows the advantages of the proposed algorithm. **(a)** For the three normal CAT properties of peak, peak-time, and area under the curve, the recommended coagulation factor concentrations from the GCM algorithm show substantial improvement in mean and standard deviation percent error for all predictions at the 24 h mark. Post-recommendation, only two samples have one property violation outside a normal range, versus actual treatment practice that violated normal ranges six times for four patients. **(b)** The number of actual CAT property violations at different times over the first 24 h for eight trauma samples compared to the total number of violations by the GCM algorithm at all of these times. **(c)** Comparison of actual coagulation factor concentrations (percent activity) at 24 h for eight trauma samples against GCM algorithm coagulation factor concentration recommendations. Coagulation factor concentrations at 24 h in all patients violate the normal coagulation factor concentration range (60–140% activity) in 38 instances, compared to zero for the GCM algorithm. **(d)** The number of actual coagulation factor concentration normal range violations at different times over the first 24 h for eight trauma samples compared to the total number of violations by the GCM algorithm at all of these times. This figure confirms the capacity of the GCM algorithm to move CAT properties toward normal while ensuring normal coagulation factor concentration levels.
